# High-Deductible Health Plans and Receipt of Guideline-Concordant Care for Adults With Chronic Illness

**DOI:** 10.1001/jamanetworkopen.2025.8045

**Published:** 2025-04-30

**Authors:** Risha Gidwani, Veronica Yank, Steven M. Asch, Lane Burgette, Aaron Kofner, Alex Peltz, Zachary Wagner

**Affiliations:** 1 Division of Health Care Policy and Research, University of Colorado School of Medicine, Aurora; 2 RAND Corporation, Santa Monica, California; 3 University of California, Los Angeles; 4 University of California, San Francisco; 5 Stanford University, Palo Alto, California; 6 RAND Corporation, Arlington, Virginia; 7 University of Southern California, Los Angeles

## Abstract

**Importance:**

High-deductible health plans (HDHPs) are a common insurance mechanism.

**Objective:**

To evaluate whether HDHPs are meeting the medical needs of persons with chronical illness.

**Design, Setting, and Participants:**

This longitudinal cohort study used 2016 to 2019 national administrative and claims data from MarketScan. Cohort members were adults aged 18 to 64 years with asthma, diabetes, hypertension, coronary artery disease, heart failure, or major depressive disorder. The treatment group was persons newly enrolling in an HDHP; the control group was persons continuously enrolled in non-HDHPs. Data were analyzed from October 2022 to April 2024, with revisions conducted between December and January 2025.

**Exposure:**

New enrollment in an HDHP was instrumented by firms changing the plan choice options to promote HDHP enrollment (ie, restricted-choice firms). Analyses used difference-in-differences models combined with this instrumental variable. Entropy balancing was used to address residual confounding.

**Main Outcomes and Measures:**

Use of annual recommended medical care, which was abstracted from disease-specific evidence-based clinical practice guidelines and included clinic visits, prescription drugs, laboratory tests, and an overall measure that combined all 3. Results were pooled across disease type to improve policy relevance of findings; disease-specific analyses were also conducted. Hypotheses were formulated prior to data collection.

**Results:**

The cohort consisted of 343 137 adults (182 532 [53.20%] female; 149 760 [43.64%] aged 55-64 years [before entropy balancing]). Groups exhibited covariate balance after entropy balancing. Restricted-choice enrollment into an HDHP was associated with reduced use of recommended medical care, with persons in HDHPs reducing their use of recommended clinic visits by 3.1 (95% CI, −4.9 to −1.2) percentage points (*P* < .001), their use of recommended prescription drugs by 9.0 (95% CI, −11.8 to −6.2) percentage points (*P* < .001), and their use of recommended annual laboratory testing by 5.7 (95% CI, −8.2 to −3.2) percentage points (*P* < .001). Overall, HDHP enrollees were 4.7 (95% CI, −6.2 to −3.3) percentage points less likely to receive recommended medical care compared with non-HDHP enrollees (*P* < .001).

**Conclusions and Relevance:**

This longitudinal cohort study of 343 137 adults with chronic illness found HDHP enrollment was associated with reduced receipt of recommended medical care across a variety of conditions. These results have important implications for recently proposed federal legislation that proposes to exempt chronic illness management from HDHP deductibles.

## Key Points

**Question:**

Do high-deductible health plans (HDHPs), a common insurance mechanism in the US, support the range of recommended medical care for individuals with chronic illness?

**Findings:**

In this cohort study of 343 137 adults using difference-in-differences models with instrumental variables and entropy balancing weights, HDHP enrollment was associated with statistically significantly lower use of evidence-based clinic, laboratory, and prescription drug care for patients, across a variety of common chronic illnesses.

**Meaning:**

These findings suggest that HDHPs may not be an appropriate insurance mechanism for individuals with chronic illness and have policy implications for recently proposed federal legislation.

## Teaser

This cohort study examines receipt of guideline-concordant health care among individuals with chronic illness enrolled in high-deductible health plans compared with those enrolled in non–high-deductible health plans.

## Introduction

High-deductible health plans (HDHPs) are a common insurance mechanism, covering 58% of privately insured people in the US.^1,2^ HDHPs have high initial cost-sharing, requiring patients to pay for 100% of most health care costs until a (high) deductible is met. In 2024, the Internal Revenue Service (IRS) defined HDHPs as those with annual deductibles of at least $1600 for an individual and $3200 for a family.^3^ However, mean deductibles can be much higher; in 2023, mean HDHP deductibles were $2418 for individuals and $4674 for families.^4^ Deductibles differ from other forms of patient cost-sharing an important way: they provide $0 coverage for care, essentially withholding insurance coverage until a high dollar threshold is met. There are concerns that the high-deductible design feature of HDHPs may discourage patients from accessing necessary medical care. Approximately half of US households of individuals younger than 65 years do not have enough liquid assets to pay the mean HDHP deductible,^5^ and almost 40% of people in the US do not have enough liquid assets to pay a $400 expense,^6^ a cost easily incurred in 1 day of health care utilization.

The purpose of health insurance is to allow persons access to necessary medical care without catastrophic expense. The seminal study in the field of cost-sharing, the RAND Health Insurance Experiment, found insurance characterized by high cost-sharing reduced use of both appropriate and inappropriate care. Notably, higher cost-sharing resulted in poorer health outcomes among low-income individuals and individuals with chronic illness.^7,8^ Much research in the 2010s found enrollment in HDHPs was associated with lower health care spending.^9,10^ More recent studies have examined the impact of HDHP enrollment on individual chronic conditions, often focused on medication use. In one study, HDHP enrollment was associated with both increased out-of-pocket costs and reduced medication adherence for patients with cardiovascular disease.^11^ However, when medications were exempt from HDHP deductibles, HDHPs were not associated with decreased pharmaceutical use.^12^

While previous quasi-experimental research indicates that HDHP enrollment was associated with reducing patients’ use of health care services,^10,13^ such studies have largely focused on 1 condition or a limited set of outcomes. In this study, we aim to provide a broader analysis of HDHPs across both a range of chronic conditions and a range of care processes recommended by the medical establishment. More than 60% of US residents are chronically ill,^14^ yet the impact of health insurance for many chronic illnesses is understudied.^15^ Persons with chronic conditions require regular care and are more likely to be top health care spenders.^16,17,18^ In this study, we used a large, national dataset to investigate the association of HDHPs with receipt of recommended medical care for people with various common chronic conditions.

This study was designed to advance the HDHP evidence in 3 important ways: it studies multiple chronic conditions, thereby increasing the policy-relevance of findings; it evaluates receipt of a range of services recommended by evidence-based clinical practice guidelines; and it does so through combining multiple quasi-experimental techniques.

## Methods

This cohort study was approved by the RAND Institutional Review Board with a waiver of informed consent due to it being a secondary analysis of existing data. This study is reported following the Strengthening the Reporting of Observational Studies in Epidemiology (STROBE) reporting guideline for cohort studies.

### Data

Our analysis uses 2016 to 2019 national MarketScan data. These data contain insurance claims submitted by 350 employers and health plans. Data from 2016 to 2019 were chosen as they represent a time frame after the switch from *International Classification of Diseases, Ninth Revision* (*ICD-9*) to *International Statistical Classification of Diseases and Related Health Problems, Tenth Revision* (*ICD-10*) coding but before the start of the COVID-19 pandemic, which was associated with a decline in elective and nonelective health care utilization.^19,20^ Data from 2016 were used for cohort identification and assessment of parallel trends only; data from 2017 represent the pre period while data from 2018 (and 2019) represent the post period in main (and sensitivity) analyses. HDHP enrollment was identified by a combination of the plan type and deductible variables, compared with IRS deductible thresholds.^21^ All plans operated on a calendar year basis.

### Cohort

Our cohort included 343 137 persons aged 18 to 64 years with chronic illness enrolled in employer-sponsored insurance who were continually enrolled in insurance for 3 years (4 years in sensitivity analyses). Almost half of the US population is enrolled in employer-sponsored insurance. We study persons with the most common conditions in the US: diabetes, hypertension, asthma, heart failure, coronary artery disease, or major depressive disorder.^14^ Chronic conditions were identified through the presence of 1 inpatient diagnosis or 2 outpatient diagnoses or procedure codes 30 days apart, using only 2016 data (eTable 1 in Supplement 1). We studied patients with 1 or 2 chronic conditions, who represent 94.3% of persons with our included chronic conditions in MarketScan data.

### Recommended Medical Care

Our main outcomes are utilization of care recommended for each chronic condition. A team including 2 physician investigators and a health economist (R.G., V.Y., S.M.A., and A.P.) extracted recommended care from evidence-based clinical practice guidelines that were applicable during the study period and used these to create programming algorithms for claims data. For each condition, we identified guidelines published by national or international entities or specialty societies that provided details on their methods and evidence base.^22,23,24,25,26,27,28,29^ Two reviewers independently extracted recommendations and then met to confirm, with disagreements resolved through discussion with a third party. Guideline recommendations that could be operationalized in claims data were included in the study (Table 1). These guidelines detail necessary care (eg, annual eye examination for individuals with known diabetic retinopathy, statin medication for individuals with known coronary artery disease), and represent the minimum level of care required. While measurement error and misclassification can never be ruled out, true deviation below these standards is likely to constitute underuse.

**Table 1.** Recommended Medical Care

Condition	Guideline category	Recommended utilization (evidence grade)	Guideline
Asthma	Clinic visit	2 Visits per y with any PCP or pulmonologist (B)	National Asthma Education and Prevention Program,^26^ 2007: “Third Expert Panel on the Diagnosis and Management of Asthma. Expert Panel Report 3: Guidelines for the Diagnosis and Management of Asthma”
Asthma	Prescription	Of 80% of the days covered by a LABA, 80% of those days also need to be covered by an inhaled corticosteroid (A)^a^	National Asthma Education and Prevention Program,^26^ 2007: “Third Expert Panel on the Diagnosis and Management of Asthma. Expert Panel Report 3: Guidelines for the Diagnosis and Management of Asthma”
Coronary artery disease	Clinic visit	1 Visit per y with any adult PCP or cardiologist (C)	Fihn et al,^23^ 2012: “2012 ACCF/AHA/ACP/AATS/PCNA/SCAI/STS guideline for the diagnosis and management of patients with stable ischemic heart disease: a report of the American College of Cardiology Foundation/American Heart Association task force on practice guidelines, and the American College of Physicians, American Association for Thoracic Surgery, Preventive Cardiovascular Nurses Association, Society for Cardiovascular Angiography and Interventions, and Society of Thoracic Surgeons”
Coronary artery disease	Prescription	≥80% Of days covered with a statin (A)	Fihn et al,^23^ 2012: “2012 ACCF/AHA/ACP/AATS/PCNA/SCAI/STS guideline for the diagnosis and management of patients with stable ischemic heart disease: a report of the American College of Cardiology Foundation/American Heart Association task force on practice guidelines, and the American College of Physicians, American Association for Thoracic Surgery, Preventive Cardiovascular Nurses Association, Society for Cardiovascular Angiography and Interventions, and Society of Thoracic Surgeons”
Coronary artery disease	Prescription	≥80% Of days covered with a β-blocker (B)	Fihn et al,^23^ 2012: “2012 ACCF/AHA/ACP/AATS/PCNA/SCAI/STS guideline for the diagnosis and management of patients with stable ischemic heart disease: a report of the American College of Cardiology Foundation/American Heart Association task force on practice guidelines, and the American College of Physicians, American Association for Thoracic Surgery, Preventive Cardiovascular Nurses Association, Society for Cardiovascular Angiography and Interventions, and Society of Thoracic Surgeons”
Diabetes	Clinic visit	Annual eye examination for patients with retinopathy (B)	American Diabetes Association,^22^ 2015: “Standards of medical care in diabetes—2015”
Diabetes	Clinic visit	1 Visit per year with any PCP or endocrinologist (B)^b^	American Diabetes Association,^22^ 2015: “Standards of medical care in diabetes—2015”
Diabetes	Laboratory testing	≥2 HbA_1c_ tests 90 d apart (E)	American Diabetes Association,^22^ 2015: “Standards of medical care in diabetes—2015”
Diabetes	Laboratory testing	eGFR annually (B)	American Diabetes Association,^22^ 2015: “Standards of medical care in diabetes—2015”
Diabetes	Laboratory testing	Urine albumin annually (B)	American Diabetes Association,^22^ 2015: “Standards of medical care in diabetes—2015”
Diabetes	Laboratory testing	Serum creatinine/eGFR and potassium if using ACE inhibitor, ARB and/or diuretic annually (E)	American Diabetes Association,^22^ 2015: “Standards of medical care in diabetes—2015”
Diabetes	Prescription	≥80% Of days covered with an ACE or ARB if the person also has hypertension (B)	American Diabetes Association,^22^ 2015: “Standards of medical care in diabetes—2015”
Heart failure	Clinic visit	1 Visit per year with any adult PCP or cardiologist (B)	Yancy et al,^29^ 2013: “2013 ACCF/AHA guideline for the management of heart failure: a report of the American College of Cardiology Foundation/American Heart Association Task Force on practice guidelines”
Heart failure	Prescription	≥80% Of days covered with an ACE or ARB for people with reduced ejection fraction (B)	Yancy et al,^29^ 2013: “2013 ACCF/AHA guideline for the management of heart failure: a report of the American College of Cardiology Foundation/American Heart Association Task Force on practice guidelines”
Heart failure	Prescription	≥80% Of days covered with a β-blocker for people with reduced ejection fraction (B)	Yancy et al,^29^ 2013: “2013 ACCF/AHA guideline for the management of heart failure: a report of the American College of Cardiology Foundation/American Heart Association Task Force on practice guidelines”
Hypertension	Clinic visit	1 Visit per year with any adult PCP or cardiologist (A)	National High Blood Pressure Education Program,^28^ 2004: *Seventh Report of the Joint National Committee on Prevention, Detection, Evaluation, and Treatment of High Blood Pressure*
Hypertension	Laboratory testing	1 laboratory test per y for serum creatine with or without eGRF (B)	National Clinical Guideline Centre (UK),^27^ 2011: *Hypertension: The Clinical Management of Primary Hypertension in Adults: Update of Clinical Guidelines 18 and 34*
MDD	Clinic visit	For all patients: 1 visit with a psychological professional, or 1 visit with a PCP where the visit has a MDD diagnosis or psychotherapy CPT code	Gelenberg et al,^24^ 2010: *Practice Guideline for the Treatment of Patients With Major Depressive Disorder*
MDD	Clinic visit	For all patients with an MDD-based prescription: 2 visits with a psychological professional, or 2 visits with a PCP where the visit has a MDD diagnosis or psychotherapy CPT code (B)	Gelenberg et al,^24^ 2010: *Practice Guideline for the Treatment of Patients With Major Depressive Disorder*
MDD	Prescription	For all patients with prescription days >0, ≥80% of days covered by antidepressants (B)	Gelenberg et al,^24^ 2010: *Practice Guideline for the Treatment of Patients With Major Depressive Disorder*

Abbreviations: ACE, angiotensin-converting enzyme; ARB, angiotensin receptor blocker; CPT, Current Procedural Terminology; eGFR, estimated glomerular filtration rate; HbA_1c_, hemoglobin A_1c_; LABA, long-acting β agonist; MDD, major depressive disorder; PCP, primary care physician.

^a^ If the patient was using a LABA for 50% of the year, and inhaled corticosteroid usage for 90% of the half year that they were using a LABA, the patient was considered to have received recommended medical care.

^b^ This is based on a recommendation that all diabetic patients have an annual foot exam. Foot exam cannot be properly ascertained in the claims data, we therefore used a PCP or endocrinologist visit to proxy the foot examination.

### Outcomes

Our primary outcome was receipt of multidimensional recommended medical care, comprising clinic visits, prescription drugs and laboratory tests annually. This was constructed as a composite outcome ranging from 0 to 1, with a denominator of the number of recommended service categories for which the patient was eligible and a numerator of the number of service categories for which the patient actually received recommended care. For example, if a person had a chronic illness for which clinic visits, prescription drugs, and laboratory tests were recommended, that person’s composite outcome would have a denominator of 3. Our approach relies on a generalization of the linear probability model.^30^ Use of a composite outcome to evaluate multidimensional quality-of-care has been used extensively in the literature.^31,32^ Use of recommended prescriptions, laboratory tests, and clinic visits were also constructed as separate secondary binary outcomes. To receive recommended laboratory or clinic care, patients had to meet the minimum number of relevant laboratory tests and outpatient visits required annually. To receive recommended drug care, patients had to have at least 80% of their days filled annually with the relevant prescriptions, an approach traditionally used in quality-of-care assessment.^33,34,35^ None of the care we study is considered preventive under the Patient Protection and Affordable Care Act; therefore, all outcomes are subject to cost-sharing.

### Statistical Analysis

We combined different quasi-experimental approaches to identify the association of HDHPs and receipt of recommended medical care. Our overarching design exploits the fact that in January 2018 some firms (employers) newly incentivized HDHP enrollment by restricting the choices of plans offered for their employees, making HDHPs more appealing. These firms are hereafter called *restricted-choice firms*. Restricted-choice firms were identified based on a variable created for this study team by the MarketScan data vendor, which measured the proportion of employees and their dependents that were enrolled in an HDHP annually. This was constructed using firm identifier (a variable available only to the data vendor). We defined a firm as having restricted choice if 0% to 35% of employees and dependents were enrolled in an HDHP in the pre period and 80% or more of employees and dependents were enrolled in an HDHP in the post period. The control group consisted of firms in which 0% to 35% of employees and dependents were enrolled in HDHPs in both the pre and post periods. We used firm-level switch to restricted choice as an instrument for HDHP enrollment, where the first stage was a difference-in-differences model that used restricted choice to estimate individual HDHP enrollment, and the second stage estimated the association between estimated individual HDHP enrollment and outcomes of interest (eMethods in Supplement 1). This approach mitigates individual selection bias, which is otherwise a concern in studies comparing people who chose to enroll in HDHPs with those who did not.^36^ Estimating our instrumental variable (IV) models within a difference-in-differences framework with a balanced panel controls for key time-invariant confounders, such as patients’ underlying preferences for seeking nonurgent medical care and baseline income level. We also estimate reduced-form models, which compare how outcomes change differently between the pre and post periods for restricted-choice and non–restricted choice firms (ie, an intention-to-treat effect). While other studies in the HDHP literature have evaluated full-replacement or restricted-choice firms,^12,13,37^ to the best of our knowledge, full-replacement or restricted-choice has not yet been used as an IV. All outcomes were assessed using linear models. Linear probability models were prioritized over logit models as our β coefficient of interest was an interaction term.^38^

We used entropy balancing to address any residual endogeneity. Entropy balancing assigns a positive weight to control group observations such that the means of relevant covariates in treatment and control groups are equivalent.^39^ Variables used for entropy balancing included comorbidity indicator variables; dual morbidity; age category; sex; geographic region; enrollment in family vs individual plan; and in the baseline year (2016), decile of outpatient visits, number of ED visits, and number of hospital visits. The latter 3 variables were used to assess baseline patient preferences for care.

#### Additional Analyses

We analyzed each chronic condition separately to assess whether the direction and size of association was consistent. In disease-specific models, entropy balancing weights were derived separately for each disease-specific cohort.

Most recommendations were based on grade A or B evidence. However, there were 4 recommendations with lower-grade evidence (Table 1). As these are widely accepted guideline recommendations within the medical community (eg, for diabetic patients, 2 hemoglobin A1c [HbA_1c_] laboratory tests ≥90 days apart [grade E]), we retained them in the main analysis, but explored the impact of excluding them in additional analyses.

#### Sensitivity Analyses

We conducted several sensitivity analyses to assess robustness of our main results. First, our main approach of instrumenting individual HDHP enrollment through presence in a restricted-choice firm prioritizes internal validity but somewhat limits external validity because many HDHP enrollees are not in a restricted-choice firm. To address this, we ran models that prioritize external validity (while reducing internal validity), eschewing IVs and using individual-level enrollment in an HDHP with an HSA (the most restrictive type of HDHP) as the main effect in a difference-in-differences model. Second, conceptually we expect the main manner through which HDHP enrollment is associated with use of recommended care is through increased cost-sharing. Model 1B uses our IV approach but also requires that the treatment group consist of anyone newly enrolled in an HDHP with an HSA. This is the highest internal validity model but has lower external validity than our main model. Model 1C also uses the IV approach but drops anyone enrolled in an HDHP in the pre period from the cohort. Model 1D clusters SEs within proxy firm identifier. As the terms of our agreement with MarketScan required firm-level identifiers be dropped from the dataset, we instead proxied firm identifiers using only variables that we know vary at the firm level and clustered SEs by the 37 proxy firm identifiers we derived. Model 1E uses the same specifications as the main model but adds in 2019 data.

*P* values were 2-sided, and statistical significance was set at α = .05. Analyses were conducted using SAS version 9.4 (SAS Institute) and Staa version 18.0 (StataCorp). Programming and analyses were conducted from October 2022 to April 2024, with revisions conducted between December and January 2025.

## Results

**Figure 1.**
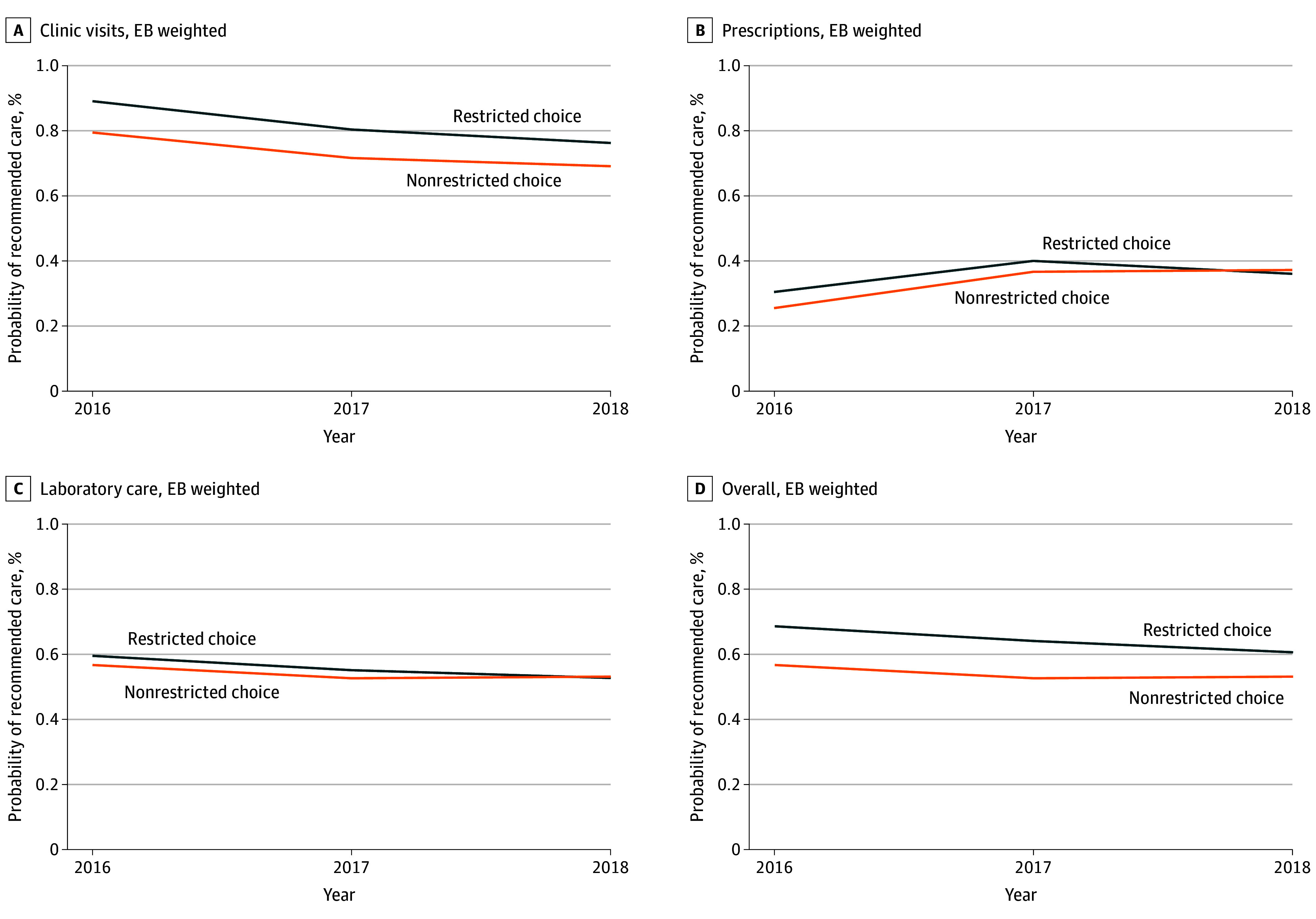
Trends in Likelihood of a Person in a Restricted Choice Firm vs Non–Restricted Choice Firm Receiving Recommended Medical Care Likelihoods are adjusted for entropy balancing (EB) weights.

Our cohort consisted of 343 137 adults (182 532 [53.2%] female; 149 760 [43.6%] aged 55-64 years [before entropy balancing]). All persons were chronically ill, with 261 575 individuals (76.2%) having 1 chronic illness and 81 562 individuals (23.8%) having 2 chronic illnesses. Cohort members had the following conditions: 242 725 individuals (70.7%) had hypertension, 101 371 individuals (29.5%) had diabetes, 31 946 individuals (9.3%) had asthma, 31 129 individuals (9.1%) had major depressive disorder, 15 576 individuals (4.5%) had coronary artery disease, and 1952 individuals (0.6%) had heart failure. After entropy balancing, groups exhibited balance on all covariates (Table 2). Figure 1 plots trends in outcomes between restricted-choice and control firms, showing that utilization trends were similar prior to the shift to restricted choice. This supports the key assumption of our difference-in-differences design: that trends in the control group are a good counterfactual for what would have happened in the treatment group in the absence of restricted choice.

**Table 2.** Demographic Characteristics of Cohort^a^

Characteristic	Before entropy balancing				After entropy balancing		
	Total No.	No. (%)		*P* value	Effective sample size, No. (%)^b^		*P* value
		Non-RCF	RCF		Non-RCF	RCF	
Total	343 137	334 168 (97.4)	8969 (2.6)	NA	189 007 (50.0)	8969 (50.0)	NA
Age, y							
18-34	28 116	27 385 (8.2)	731 (8.2)	<.001	15 404 (8.2)	731 (8.2)	>.99
35-44	50 557	49 665 (14.9)	892 (10)		18 806 (10.0)	892 (10.0)	
45-54	114 704	112 053 (33.5)	2651 (29.6)		55 870 (29.6)	2651 (29.6)	
55-64	149 760	145 065 (43.4)	4695 (52.4)		98 945 (52.4)	4695 (52.4)	
Sex							
Male	160 605	155 531 (46.5)	5074 (56.6)	<.001	106 921 (56.6)	5074 (56.6)	>.99
Female	182 532	178 637 (53.5)	3895 (43.4)		82 086 (43.4)	3895 (43.4)	
Region							
Northeast	41 094	39 254 (11.8)	1840 (20.5)	<.001	38 784 (20.5)	1840 (20.5)	>.99
North Central	79 956	79 891 (23.9)	65 (0.7)		1361 (0.7)	65 (0.7)	
South	189 156	183 844 (55)	5312 (59.2)		111 949 (59.2)	5312 (59.2)	
West	28 602	26 852 (8.0)	1750 (19.5)		36 875 (19.5)	1750 (19.5)	
Unknown	4329	4327 (1.3)	2 (<0.1)		38 (<0.1)	2 (<0.1)	
Plan type							
Individual	122 356	120 414 (36.0)	1942 (21.7)	<.001	40 920 (21.7)	1942 (21.7)	>.99
Family	220 781	213 754 (64.0)	7027 (78.4)		148 087 (78.4)	7027 (78.4)	
Chronic conditions, No.							
1	261 575	254 723 (76.2)	6852 (76.4)	.71	144 401 (76.4)	6852 (76.4)	>.99
2	81 562	79 445 (23.8)	2117 (23.6)		44 606 (23.6)	2117 (23.6)	
Coronary artery disease	15 576	15 135 (4.5)	441 (4.9)	.08	9299 (4.9)	441 (4.9)	>.99
Hypertension	242 725	236 575 (70.8)	6150 (68.6)	<.001	129 602 (68.6)	6150 (68.6)	>.99
Heart Failure	1952	1907 (0.6)	45 (0.5)	.39	945 (0.5)	45 (0.5)	>.99
Asthma	31 946	30 985 (9.3)	961 (10.7)	<.001	20 243 (10.7)	961 (10.7)	>.99
Major depressive disorder	31 129	30 202 (9.0)	927 (10.3)	<.001	19 543 (10.3)	927 (10.3)	>.99
Diabetes	101 371	98 809 (29.6)	2562 (28.6)	.04	53 999 (28.6)	2562 (28.6)	>.99

Abbreviations: NA, not applicable; RCF, restricted-choice firm.

^a^ Data shown are from 2016. The cohort contains a balanced panel over time.

^b^ Effective sample size applies to the control group only since treated observations are unweighted and reflects the impact of entropy balancing weights.

People with chronic conditions used significantly less recommended medical care across all outcome categories after their firm switched to restricted choice (Table 3). Enrollees in restricted choice firms had 52.0 (95% CI, 50.9 to 53.0) percentage points increased likelihood of being enrolled in an HDHP relative to the control group. *F*-statistics ranged from 3477 to 9512, depending on the subcohort used (Table 3), indicating restricted-choice firm was a strong instrument for HDHP enrollment.

**Table 3.** Results Operationalized Using Instrumented Difference-in-Difference Models With Entropy Balancing Weights^a^

Model	Care received			
	Clinic visit	Prescription drug	Laboratory testing	Composite measure
Association of RCF with HDHP enrollment^a^^,^^b^				
Estimate (95% CI)	0.520 (0.509 to 0.530)	0.512 (0.495 to 0.529)	0.508 (0.496 to 0.519)	0.520 (0.509 to 0.530)
*P* value	<.001	<.001	<.001	<.001
*F*-Statistic^c^	9511.85	3477.19	7462.94	9511.85
Association of RCF with outcome^b^^,^^d^				
Estimate (95% CI)	−0.016 (−0.026 to −0.006)	−0.046 (−0.060 to −0.031)	−0.029 (−0.042 to −0.016)	−0.025 (−0.032 to −0.017)
*P* value	<.001	<.001	<.001	<.001
Association of HDHP with outcome^e^				
Estimate (95% CI)	−0.031 (−0.049 to −0.012)	−0.090 (−0.118 to −0.062)	−0.057 (−0.082 to −0.032)	−0.047 (−0.062 to −0.033)
*P* value	<.001	<.001	<.001	<.001
Individuals, No.				
Total	343 137	126 617	288 745	343 137
Treatment	8969	3452	7369	8969
Control	334 168	123 165	281 376	334 168
Predicted probability of receiving care^f^				
HDHP, estimate (95% CI), %	72.1 (70.7 to 73.5)	30.9 (28.7 to 33.0)	49.1 (47.2 to 52.4)	56.8 (55.7 to 57.9)
Non-HDHP, estimate (95% CI), %	75.2 (74.6 to 75.8)	39.8 (38.9 to 40.8)	54.9 (54.1 to 55.7)	61.6 (61.1 to 62.1)

Abbreviations: HDHP, high-deductible health plan; RCF, restricted choice firm.

^a^ Probability of newly enrolling into an HDHP as estimated by a firm switching to restricted choice.

^b^ Estimated using difference-in-difference models that interact RCF with the time variable (post).

^c^ *F*-statistics differ across clinic visit, prescription drug and laboratory testing outcomes, as the sample used for each of these outcomes varies (eg, all persons in the cohort were eligible for a clinic visit, but not all persons in the cohort are eligible for prescription drugs).

^d^ Change in mean use of recommended care as estimated by a firm switching to restricted choice.

^e^ Change in mean use of recommended care as estimated by HDHP. Estimated using an instrumental variables regression where restricted choice is used as an instrument for individual-level HDHP enrollment.

^f^ Probabilities of received care are estimated from the IV difference-in-differences models and represent the absolute likelihood of receiving care if in an HDHP. All *P* < .05.

### Main Results

Results from difference-in-differences models using IVs show that enrollment into an HDHP was associated with declines in use of recommended medical care across all care categories (Table 3). Persons with chronic illness in HDHPs reduced their use of recommended clinic visits by 3.1 (95% CI, −4.9 to −1.2) percentage points compared with those in non-HDHPs (*P* < .001). HDHP enrollees were 9.0 (95% CI −11.8 to −6.2) percentage points less likely to have received recommended drug treatment (*P* < .001). HDHP enrollees also reduced their use of recommended annual laboratory testing by 5.7 (95% CI, −8.2 to −3.2) percentage points compared with non-HDHP enrollees (*P* < .001). Evaluations of the composite outcome showed that HDHP enrollees with chronic illness were 4.7 (95% CI, −6.2 to −3.3) percentage points less likely to receive overall recommended medical care than those in non-HDHPs (*P* < .001). Predicted probabilities generated from our IV models indicate that persons enrolled in HDHPs receiving medical care, compared with non-HDHP enrollees, were less likely to receive recommended clinic visits (72.1% vs 75.2%), drug treatment (30.9% vs 39.8%), laboratory testing (49.1% vs 54.9%), and overall medical care (56.8% vs 61.6%).

**Figure 2.**
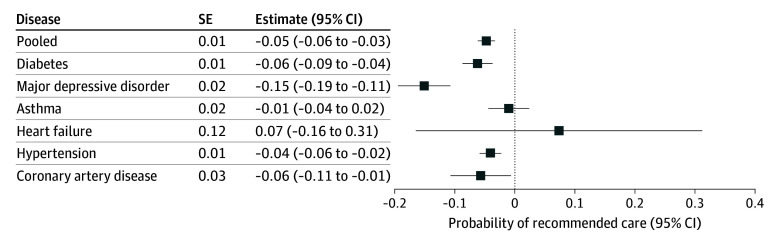
Difference in Likelihood of Receiving Recommended Medical Care by Disease Type for Persons Enrolled in High-Deductible Health Plans vs Non–High-Deductible Health Plans Analyses based on regressions that instrument high-deductible health plan enrollment using employment in a restricted choice firm. The outcome presented is the composite measure of overall recommended medical care.

Figure 2 shows model results for the composite outcome in the form of a forest plot, with 1 estimate per disease type. The direction of association is consistent across disease types, with the exception of heart failure. Results were not statistically significant for asthma or heart failure; the latter was underpowered with wide CIs. Results were strongest for major depressive disorder.

### Sensitivity Analyses

Results from other sensitivity analyses revealed no change in direction of association and virtually no change in significance. When excluding 4 recommendations with grade C or E evidence, results remained the same, with no change in direction or significance and β coefficients that changed only in the thousandths place (eTable 5 in Supplement 1). Model 1A found the same direction of associations with HDHPs in all 4 outcomes as the main model, with results remaining statistically significant, supporting the external validity of our main results. β coefficients were slightly muted (eTable 6 in Supplement 1). In both Model 1B and Model 1C, there was no change with respect to direction or significance of results and virtually no change to β coefficients. Model 1D, which clustered observations within proxy firm identifier, found no change with respect to magnitude or direction of association; however, in this analysis, the findings for HDHPs and clinic visits lost statistical significance (all 3 other outcomes remained significant). Model 1E, which added 2019 data to the post period, also found no change with respect to direction or significance of results and similar β coefficients as the main model.

## Discussion

Results from our cohort study, which focuses on the most common chronic conditions in the US, indicate that HDHPs were associated with reduced access to medical care across multiple dimensions, including clinic visits, drugs, and recommended laboratory testing. Insurance is the gatekeeper through which most people in the US access health care. Persons with chronic illness, in particular, require regular access to medical care. Given the prevalence of chronic illness in this country, this translates into a large number of people negatively affected by enrollment in HDHPs. In 2018, there were 3 056 295 persons in MarketScan data with 1 of the chronic illnesses we studied. If 4.7% fewer of them receive recommended medical care, that corresponds to 143 646 fewer people each year in this dataset alone not receiving care required for chronic illness. The levels of care that we study are minimal standards—for example, 1 visit a year to a medical professional for a patient with diagnosed heart failure or 1 eye examination a year for a patient with diabetes and diagnosed retinopathy. That such basic levels of care are less likely to be met when persons enroll in HDHPs engenders concern.

It is challenging to compare HDHP studies because group definitions vary widely. Some define HDHPs as plans with deductibles as low as $1000,^40^ while others identify HDHPs where employer contributions render the effective deductible to be $0.^41^ Studies that use full-replacement firms use varying HDHP enrollment thresholds, some as low as 70% or 73%.^42,43^ Additionally, some studies exclude any pre period HDHP enrollment, while others allow it.^13,43^ Such inconsistency complicates cross-study comparisons. Nonetheless our work adds to a robust literature indicating that enrollment in HDHPs is associated with in lower health care utilization.^10,13,44^ We studied, across multiple chronic conditions, care recommended vs care received. Our results indicate that HDHP enrollment was associated with a reduction in care, such that persons of numerous illness profiles were not receiving the minimum level of medical care recommended to manage their disease. Sensitivity analyses found that these results were robust to a number of model specifications. The sole exception to this was for clinic visits: models with SEs clustered within proxy firm identifiers showed no significant results for outpatient care, although remained significant for recommended drugs, laboratory tests, and overall care.

Our work also adds to the literature on the association between health insurance and chronic illness.^15,45^ Our findings indicating lower receipt of recommended medical care, as well as other work indicating that higher cost-sharing for services for persons with chronic illness is associated with increases in emergency department visits or mortality,^15,46,47,48^ together suggest that reductions in use of recommended medical care due to insurance-based cost-sharing will have commensurate negative effects on a chronically ill population.

Our work has direct implications for federal legislation. The bipartisan Chronic Disease Management Act of 2021, currently under review in Congress, proposes to exempt services that are low-cost and effective in treating chronic disease from the deductibles of HDHPs.^49^ Our work suggests that access to evidence-based recommended medical care should be considered in the pool of candidate services and that major depressive disorder should be included in the list of chronic illnesses.

### Limitations

This study has some limitations. Our results might not generalize to full populations enrolled in HDHPs. First, our data come from one of the largest sources of claims data for persons with employer-sponsored insurance in the US, including 25 million persons per year; however, the data do not contain the full population of persons with such insurance. We also required cohort members to be continuously enrolled in insurance for 3 years. However, our cohort does represent the population of persons with employer-sponsored insurance in the MarketScan data, with proportions of comorbidities similar to what is seen in the overall dataset. The exception to this was major depressive disorder, which our cohort was less likely to have than the overall MarketScan population. We found largest directions of HDHP associations for persons with major depressive disorder, suggesting that an even larger number of people would be negatively affected by HDHP enrollment than in the rough calculations we present. Second, our IV approach estimates a local average treatment effect, meaning the association of HDHP enrollment for persons who chose HDHPs under a restricted choice set who would otherwise have remained in a non-HDHP plan. Results from models using this IV may not generalize to persons who would have always chosen an HDHP or who would have never chosen an HDHP under any circumstance. However, that our sensitivity analysis using individual-level enrollment in HDHPs (model 1A) which did not use an IV approach, also showed significant reductions in use of all care indicates that the generalizability of our results may not be restricted only to individuals who chose the HDHP. Furthermore, our outcomes consisted of care practices that were present in evidence-based clinical practice guidelines and could be measured through administrative data. Some recommendations could not be translated into coding algorithms suitable for claims data, such as achieving a specific level of diastolic blood pressure. Thus, while our composite measure evaluates clinical care, prescription drugs, and laboratory tests, it is not a fully comprehensive measure of recommended medical care, and our estimates about the ability of HDHPs to connect patients to necessary medical care may therefore be conservative.

## Conclusions

The findings of this cohort study indicate that while HDHPs and chronic illness are both highly prevalent in the US, they may not be appropriate bedfellows. All individuals with chronic illness require a minimum level of care to manage their illness, including at least 1 visit a year to a medical professional, and laboratory tests and/or prescription drugs as appropriate. We found that HDHP enrollment was associated with a lower use of basic levels of recommended medical care for persons with chronic illness, a finding that may be informative to recently proposed federal legislation.

## Notes

Supplement 1.
eMethods.
**eTable 1.** *ICD-10* Codes Used to Identify Chronic Conditions **eTable 2.** Recommended Medical Care: 2 Conditions **eTable 3.** CPT Codes Used to Assess Office Visits for Chronic Illness Management **eTable 4.** Full Model Results, Recommended Medical Care **eTable 5.** Model Results, Operationalized Using Instrumented Difference-in-difference Models with Entropy Balancing Weights, Excluding 4 Care Recommendations With Grade C or E Evidence **eTable 6.** Main Results From Sensitivity Analyses, Models 1A-1E

Supplement 2.
Data Sharing Statement
