# Supplementary material for: High-Deductible Health Plans and Receipt of Guideline-Concordant Care for Adults With Chronic Illness
Source: JAMA Netw Open. 2025 Apr 30;8(4):e258045. doi: 10.1001/jamanetworkopen.2025.8045 (PMC12044513; doi:10.1001/jamanetworkopen.2025.8045)
Supplement: Supplement 1. — eMethods. eTable 1. ICD-10 Codes Used to Identify Chronic Conditions eTable 2. Recommended Medical Care: 2 Conditions eTable 3. CPT Codes Used to Assess Office Visits for Chronic Illness Management eTable 4. Full Model Results, Recommended Medical Care eTable 5. Model Results, Operationalized Using Instrumented Difference-in-difference Models with Entropy Balancing Weights, Excluding 4 Care Recommendations With Grade C or E Evidence eTable 6. Main Results From Sensitivity Analyses, Models 1A-1E [file jamanetwopen-e258045-s001.pdf]

Supplemental Online Content

Gidwani R, Yank V, Asch SM, et al. High-deductible health plans and receipt of guideline-concordant care for adults with chronic illness. *JAMA Netw Open*. 2025;8(4):e258045. doi:10.1001/jamanetworkopen.2025.8045

eMethods.
eTable 1. *ICD-10* Codes Used to Identify Chronic Conditions
eTable 2. Recommended Medical Care: 2 Conditions
eTable 3. CPT Codes Used to Assess Office Visits for Chronic Illness Management
eTable 4. Full Model Results, Recommended Medical Care
eTable 5. Model Results, Operationalized Using Instrumented Difference-in-difference Models with Entropy Balancing Weights, Excluding 4 Care Recommendations With Grade C or E Evidence
eTable 6. Main Results From Sensitivity Analyses, Models 1A-1E

This supplemental material has been provided by the authors to give readers additional information about their work.

© 2025 Gidwani R et al. *JAMA Network Open*.

Statistical Analysis Plan

Recommended Medical Care

Our study outcomes are based on utilization of guideline-recommended care for each chronic condition. The guideline sub team, which included physician investigators and health services researchers (VY, SA, RG, AP) identified pertinent guidelines published by national or international expert bodies, specialty societies, or professional associations in 2015 or earlier. This time frame reflects the evidence available during the period of time in which outcomes were assessed (2016-2018). Eligible guidelines were those that described the methods, evidence base, and evidence grades for their recommendation(s).[1-8] While evidence grading systems varied somewhat across guidelines, A and B grades consistently indicated evidence from well-conducted randomized controlled trials, meta-analyses, and non-randomized studies (cohort, case control), and C and E grades indicated evidence from consensus opinion of experts, case studies, or standard of care.

Two reviewers independently identified and extracted recommended medical care from guidelines and then met to confirm agreement and data accuracy, with disagreements resolved through discussions with a third party. The guideline team also determined whether the MarketScan data set could address the care recommendation. Care recommendations that could be operationalized with MarketScan data were included in the study (see eTable 2a and eTable 2b). For example, guidelines that required an assessment of clinical status, such as diastolic and systolic blood pressure, were excluded as they could not be assessed using claims data.

We implemented the assessment of 19 disease-specific recommendations across 6 conditions: asthma, hypertension, diabetes, coronary artery disease, heart failure, and major depressive disorder. Of these, 16 had an A or B evidence grade, 3 had a C or E evidence grade.

A patient was considered to have the relevant clinic visit if they had an evaluation and management (E&M) visit to the relevant provider type for their disease, with the latter identified by the STDPROV variable in the dataset. For example, for a diabetes visit, patients had to have an E&M CPT code denoted during an office visit to a primary care physician or an endocrinologist. The only exception to this was for major depressive disorder (MDD), for which the patient also had to have an MDD diagnosis in addition to an E&M code if the visit was to a PCP. Providers classified in the dataset as Psychiatrics, Psychologists, or Pyschiatric Nurses were considered psychological professionals.

In cases where recommendations denoted a frequency range for care (e.g., patients should be seen between 2 to 4 times per year), we used the lower bound estimate to determine recommended medical care (e.g., 2 visits per year).

Calculating Days' Supply of Prescription Drugs

The number of days a prescription was filled was assessed by first creating a 3-year daily dataset covering 2016, 2017 and 2018. In this 1096-day dataset, each day was assigned a drug-filled value of 0 or 1 by looking at the day the prescription was filled, and the number of days' supply it represented. For example, if a prescription was filled on January 15, 2016, with a 90-day supply, days 15-105 were assigned a value of 1. Data were then collapsed to the year level (2016, 2017, or 2018) and the proportion of days covered by a prescription were then assessed. Of note, 2016 data were used only to assess parallel trends assumptions and did not enter into the statistical models. To address any potential issues of stockpiling, we used the following approach: if a person had a 90-day prescription filled on Dec 2, 2017, 30 days of that prescription were allocated to 2017 and the remaining 60 days were allocated to 2018. This approach assigns the prescription to the year in, and therefore the insurance plan through,

© 2025 Gidwani R et al. *JAMA Network Open*.

which the prescription was first filled. This represents a conservative approach, and may mute the size of the prescription drug beta-coefficient for HDHP enrollment. Proportions of 80% or higher were considered as having met the quality-indicator for prescription drugs filled.

This "proportion of days covered" specification as chosen as the research question of interest was how insurance plan type affects receipt of prescription drugs.[9] An alternative specification was chosen in which days' supply was only allocated to the year in which the prescription occurred and any days that spilled over into the subsequent year were disregarded. This results in estimates with no or minimal difference, with the exception of major depressive disorder, for which using the alternative specification increased the proportion of persons with recommended drugs by 6 percentage points. However, as the same degree of increase occurred across both the treatment and control groups, the resulting relative estimates are unaffected.

Multi-Morbid Patients

In cases where patients had dual morbidities (25.64% of our sample), we required them to receive the sum of their laboratory and prescription drug requirements across the two conditions, but did not require them to receive the sum of their clinical care requirements. Rather, we assumed that a primary care physician (defined as an internal medicine physician, family practice physician or geriatrician) could treat two conditions in one visit. This is grounded in evidence that a U.S. PCP treats, on average, 3.05 problems per visit.[10] For drugs, we required that they achieve at least 80% days filled for both conditions. For example, if a patient had both diabetes and hypertension, they had to have 80% of their days filled for their diabetes medications as well as 80% of their days filled for their hypertensive medications to be considered having met the standard of care for drugs.

Services Excluded in Sensitivity Analyses

We excluded four services that had Grade C or E evidence in sensitivity analyses. These services are: 1) 1 visit per year with any adult primary care physician or cardiologist for patients with coronary artery disease (grade C evidence); 2) at least 2 hemoglobin A1c tests 90 days apart for patients with diabetes (grade E evidence); 3) serum creatinine and estimated glomerular filtration rate (eGFR) labs annually for patients with diabetes who are on angiotensin-converting enzyme inhibitors (ACE-I) or angiotensin receptor blockers (ARBs) annually (grade E evidence); and 4) potassium labs annually for patients with diabetes who are on ACE-Is or ARBs annually (grade e evidence).

Empirical Approach

Our analytical approach exploits the fact that some employers in the MarketScan data switch from offering a range of insurance options (i.e. HDHPs and non-HDHPs) to offering mostly HDHPs as an insurance option. We refer to the employers, or firms, that make this switch as restricted choice firms (RCFs). The difference-in-differences and instrumental variables design for which we report results in the main text are described in detail below.

First, we used a difference-in-differences model to compare changes in outcomes between persons whose firm switched to a RCF vs. persons whose firms that did not switch to restricted choice. This model used the following regression specification:

© 2025 Gidwani R et al. *JAMA Network Open*.

Difference-in-Differences (Reduced Form)

(1) $$Y_{it} = \beta_0 + \beta_1 RCF_i + \beta_2 Post_t + \beta_3 RCF_i \times Post_t + X_{it}\beta_4 + \epsilon_{it}$$

Where Y_{it} is the outcome for employee i at time t, RCF indicates whether employee i was part of a firm that switched to restricted choice, $Post$ is an indicator for whether the time period is after the switch to restricted choice (January 2018), and $RCF \times Post$ indicates whether the employee is part of a restricted choice firm and the switch to restricted choice had already occurred. The term $\boldsymbol{X_{it}}$ is a vector of covariates including indicator variables for each comorbidity, whether the individual had single or dual morbidity, enrollment in a family vs. individual health insurance plan, age group category, geographic region and sex. We also use entropy balancing weights to adjust ensure baseline balance on all the aforementioned covariates. The main coefficient of interest in this regression framework is β_3, which estimates how outcomes changed differently from the pre-period to the post-period between employees in RCFs versus non-RCFs. This is the beta coefficient we report in Table 3 of the main paper (reduced form models results). For β_3 to be interpreted as causal, the assumption that the outcomes of non-RCF employees would have changed in the same way as the RCF firm employees, had restricted choice not occurred, must hold.

Ultimately, our interest is in how HDHP enrollment (rather than obtaining insurance through a restricted choice firm) affects our outcomes of interest. We therefore used an instrumental variables approach and estimated a two-stage least squares model outlined below in equations 2 and 3.

First Stage

(2) $$HDHP_{it} = \alpha_0 + \alpha_1 RCF_i + \alpha_2 Post_t + \alpha_3 RCF_i \times Post_t + X_{it}\alpha_4 + \epsilon_{it}$$

Second Stage

(3) $$Y_{it} = \beta_0 + \beta_1 RCF_i + \beta_2 Post_t + \beta_3 \widehat{HDHP}_{it} + X_{it}\beta_4 + \epsilon_{it}$$

In the first stage equation (equation 2), α_3 estimates the extent to which being part of a RCF led to a person newly enrolled into a HDHP in the post period (we report these coefficients in the first row of Table 3 in the main paper). In the second stage, predicted HDHP enrollment ($\widehat{HDHP}_{it}$) estimated from the first stage is used directly as a covariate in the main outcomes regression. Thus, this estimates the effect of new HDHP enrollment on outcomes. Standard errors were clustered at the individual level in all models.

© 2025 Gidwani R et al. *JAMA Network Open*.

Table A1: ICD-10 Codes Used to Identify Chronic Conditions

Condition	Code	Code Descriptions
Diabetes	E10.10	Type 1 diabetes mellitus with ketoacidosis without coma
Diabetes	E10.11	Type 1 diabetes mellitus with ketoacidosis with coma
Diabetes	E10.21	Type 1 diabetes mellitus with diabetic nephropathy
Diabetes	E10.22	Type 1 diabetes mellitus with diabetic chronic kidney disease
Diabetes	E10.29	Type 1 diabetes mellitus with other diabetic kidney complication
Diabetes	E10.311	Type 1 diabetes mellitus with unspecified diabetic retinopathy with macular edema
Diabetes	E10.319	Type 1 diabetes mellitus with unspecified diabetic retinopathy without macular edema
Diabetes	E10.321	Type 1 diabetes mellitus with mild non-proliferative diabetic retinopathy with macular edema
Diabetes	E10.3211	Type 1 diabetes mellitus with mild non-proliferative diabetic retinopathy with macular edema, right eye
Diabetes	E10.3212	Type 1 diabetes mellitus with mild non-proliferative diabetic retinopathy with macular edema, left eye
Diabetes	E10.3213	Type 1 diabetes mellitus with mild non-proliferative diabetic retinopathy with macular edema, bilateral
Diabetes	E10.3219	Type 1 diabetes mellitus with mild non-proliferative diabetic retinopathy with macular edema, unspecified eye
Diabetes	E10.329	Type 1 diabetes mellitus with mild non-proliferative diabetic retinopathy without macular edema
Diabetes	E10.3291	Type 1 diabetes mellitus with mild non-proliferative diabetic retinopathy without macular edema, right eye
Diabetes	E10.3292	Type 1 diabetes mellitus with mild non-proliferative diabetic retinopathy without macular edema, left eye
Diabetes	E10.3293	Type 1 diabetes mellitus with mild non-proliferative diabetic retinopathy without macular edema, bilateral
Diabetes	E10.3299	Type 1 diabetes mellitus with mild non-proliferative diabetic retinopathy without macular edema, unspecified eye
Diabetes	E10.331	Type 1 diabetes mellitus with moderate non-proliferative diabetic retinopathy with macular edema
Diabetes	E10.3311	Type 1 diabetes mellitus with moderate non-proliferative diabetic retinopathy with macular edema, right eye
Diabetes	E10.3312	Type 1 diabetes mellitus with moderate non-proliferative diabetic retinopathy with macular edema, left eye
Diabetes	E10.3313	Type 1 diabetes mellitus with moderate non-proliferative diabetic retinopathy with macular edema, bilateral
Diabetes	E10.3319	Type 1 diabetes mellitus with moderate non-proliferative diabetic retinopathy with macular edema, unspecified eye
Diabetes	E10.339	Type 1 diabetes mellitus with moderate non-proliferative diabetic retinopathy without macular edema

© 2025 Gidwani R et al. *JAMA Network Open*.

Condition	Code	Code Descriptions
Diabetes	E10.3391	Type 1 diabetes mellitus with moderate non-proliferative diabetic retinopathy without macular edema, right eye
Diabetes	E10.3392	Type 1 diabetes mellitus with moderate non-proliferative diabetic retinopathy without macular edema, left eye
Diabetes	E10.3393	Type 1 diabetes mellitus with moderate non-proliferative diabetic retinopathy without macular edema, bilateral
Diabetes	E10.3399	Type 1 diabetes mellitus with moderate non-proliferative diabetic retinopathy without macular edema, unspecified eye
Diabetes	E10.341	Type 1 diabetes mellitus with severe non-proliferative diabetic retinopathy with macular edema
Diabetes	E10.3411	Type 1 diabetes mellitus with severe non-proliferative diabetic retinopathy with macular edema, right eye
Diabetes	E10.3412	Type 1 diabetes mellitus with severe non-proliferative diabetic retinopathy with macular edema, left eye
Diabetes	E10.3413	Type 1 diabetes mellitus with severe non-proliferative diabetic retinopathy with macular edema, bilateral
Diabetes	E10.3419	Type 1 diabetes mellitus with severe non-proliferative diabetic retinopathy with macular edema, unspecified eye
Diabetes	E10.349	Type 1 diabetes mellitus with severe non-proliferative diabetic retinopathy without macular edema
Diabetes	E10.3491	Type 1 diabetes mellitus with severe non-proliferative diabetic retinopathy without macular edema, right eye
Diabetes	E10.3492	Type 1 diabetes mellitus with severe non-proliferative diabetic retinopathy without macular edema, left eye
Diabetes	E10.3493	Type 1 diabetes mellitus with severe non-proliferative diabetic retinopathy without macular edema, bilateral
Diabetes	E10.3499	Type 1 diabetes mellitus with severe non-proliferative diabetic retinopathy without macular edema, unspecified eye
Diabetes	E10.351	Type 1 diabetes mellitus with proliferative diabetic retinopathy with macular edema
Diabetes	E10.3511	Type 1 diabetes mellitus with proliferative diabetic retinopathy with macular edema, right eye
Diabetes	E10.3512	Type 1 diabetes mellitus with proliferative diabetic retinopathy with macular edema, left eye
Diabetes	E10.3513	Type 1 diabetes mellitus with proliferative diabetic retinopathy with macular edema, bilateral
Diabetes	E10.3519	Type 1 diabetes mellitus with proliferative diabetic retinopathy with macular edema, unspecified eye
Diabetes	E10.3521	Type 1 diabetes mellitus with proliferative diabetic retinopathy with traction retinal detachment involving the macula, right eye
Diabetes	E10.3522	Type 1 diabetes mellitus with proliferative diabetic retinopathy with traction retinal detachment involving the macula, left eye

© 2025 Gidwani R et al. *JAMA Network Open*.

Condition	Code	Code Descriptions
Diabetes	E10.3523	Type 1 diabetes mellitus with proliferative diabetic retinopathy with traction retinal detachment involving the macula, bilateral
Diabetes	E10.3529	Type 1 diabetes mellitus with proliferative diabetic retinopathy with traction retinal detachment involving the macula, unspecified eye
Diabetes	E10.3531	Type 1 diabetes mellitus with proliferative diabetic retinopathy with traction retinal detachment not involving the macula, right eye
Diabetes	E10.3532	Type 1 diabetes mellitus with proliferative diabetic retinopathy with traction retinal detachment not involving the macula, left eye
Diabetes	E10.3533	Type 1 diabetes mellitus with proliferative diabetic retinopathy with traction retinal detachment not involving the macula, bilateral
Diabetes	E10.3539	Type 1 diabetes mellitus with proliferative diabetic retinopathy with traction retinal detachment not involving the macula, unspecified eye
Diabetes	E10.3541	Type 1 diabetes mellitus with proliferative diabetic retinopathy with combined traction retinal detachment and rhegmatogenous retinal detachment, right eye
Diabetes	E10.3542	Type 1 diabetes mellitus with proliferative diabetic retinopathy with combined traction retinal detachment and rhegmatogenous retinal detachment, left eye
Diabetes	E10.3543	Type 1 diabetes mellitus with proliferative diabetic retinopathy with combined traction retinal detachment and rhegmatogenous retinal detachment, bilateral
Diabetes	E10.3549	Type 1 diabetes mellitus with proliferative diabetic retinopathy with combined traction retinal detachment and rhegmatogenous retinal detachment, unspecified eye
Diabetes	E10.3551	Type 1 diabetes mellitus with stable proliferative diabetic retinopathy, right eye
Diabetes	E10.3552	Type 1 diabetes mellitus with stable proliferative diabetic retinopathy, left eye
Diabetes	E10.3553	Type 1 diabetes mellitus with stable proliferative diabetic retinopathy, bilateral
Diabetes	E10.3559	Type 1 diabetes mellitus with stable proliferative diabetic retinopathy, unspecified eye
Diabetes	E10.359	Type 1 diabetes mellitus with proliferative diabetic retinopathy without macular edema
Diabetes	E10.3591	Type 1 diabetes mellitus with proliferative diabetic retinopathy without macular edema, right eye
Diabetes	E10.3592	Type 1 diabetes mellitus with proliferative diabetic retinopathy without macular edema, left eye
Diabetes	E10.3593	Type 1 diabetes mellitus with proliferative diabetic retinopathy without macular edema, bilateral
Diabetes	E10.3599	Type 1 diabetes mellitus with proliferative diabetic retinopathy without macular edema, unspecified eye
Diabetes	E10.36	Type 1 diabetes mellitus with diabetic cataract

© 2025 Gidwani R et al. *JAMA Network Open*.

Condition	Code	Code Descriptions
Diabetes	E10.37X1	Type 1 diabetes mellitus with diabetic macular edema, resolved following treatment, right eye
Diabetes	E10.37X2	Type 1 diabetes mellitus with diabetic macular edema, resolved following treatment, left eye
Diabetes	E10.37X3	Type 1 diabetes mellitus with diabetic macular edema, resolved following treatment, bilateral
Diabetes	E10.37X9	Type 1 diabetes mellitus with diabetic macular edema, resolved following treatment, unspecified eye
Diabetes	E10.39	Type 1 diabetes mellitus with other diabetic ophthalmic complication
Diabetes	E10.40	Type 1 diabetes mellitus with diabetic neuropathy, unspecified
Diabetes	E10.41	Type 1 diabetes mellitus with diabetic mononeuropathy
Diabetes	E10.42	Type 1 diabetes mellitus with diabetic polyneuropathy
Diabetes	E10.43	Type 1 diabetes mellitus with diabetic autonomic (poly)neuropathy
Diabetes	E10.44	Type 1 diabetes mellitus with diabetic amyotrophy
Diabetes	E10.49	Type 1 diabetes mellitus with other diabetic neurological complication
Diabetes	E10.51	Type 1 diabetes mellitus with diabetic peripheral angiopathy without gangrene
Diabetes	E10.52	Type 1 diabetes mellitus with diabetic peripheral angiopathy with gangrene
Diabetes	E10.59	Type 1 diabetes mellitus with other circulatory complications
Diabetes	E10.610	Type 1 diabetes mellitus with diabetic neuropathic arthropathy
Diabetes	E10.618	Type 1 diabetes mellitus with other diabetic arthropathy
Diabetes	E10.620	Type 1 diabetes mellitus with diabetic dermatitis
Diabetes	E10.621	Type 1 diabetes mellitus with foot ulcer
Diabetes	E10.622	Type 1 diabetes mellitus with other skin ulcer
Diabetes	E10.628	Type 1 diabetes mellitus with other skin complications
Diabetes	E10.630	Type 1 diabetes mellitus with periodontal disease
Diabetes	E10.638	Type 1 diabetes mellitus with other oral complications
Diabetes	E10.641	Type 1 diabetes mellitus with hypoglycemia with coma
Diabetes	E10.649	Type 1 diabetes mellitus with hypoglycemia without coma
Diabetes	E10.65	Type 1 diabetes mellitus with hyperglycemia
Diabetes	E10.69	Type 1 diabetes mellitus with other specified complication
Diabetes	E10.8	Type 1 diabetes mellitus with unspecified complications
Diabetes	E10.9	Type 1 diabetes mellitus without complications
Diabetes	E11.00	Type 2 diabetes mellitus with hyperosmolarity without nonketotic hyperglycemic-hyperosmolar coma (NKHHC)
Diabetes	E11.01	Type 2 diabetes mellitus with hyperosmolarity with coma
Diabetes	E11.21	Type 2 diabetes mellitus with diabetic nephropathy

© 2025 Gidwani R et al. *JAMA Network Open*.

Condition	Code	Code Descriptions
Diabetes	E11.22	Type 2 diabetes mellitus with diabetic chronic kidney disease
Diabetes	E11.29	Type 2 diabetes mellitus with other diabetic kidney complication
Diabetes	E11.311	Type 2 diabetes mellitus with unspecified diabetic retinopathy with macular edema
Diabetes	E11.319	Type 2 diabetes mellitus with unspecified diabetic retinopathy without macular edema
Diabetes	E11.321	Type 2 diabetes mellitus with mild non-proliferative diabetic retinopathy with macular edema
Diabetes	E11.3211	Type 2 diabetes mellitus with mild non-proliferative diabetic retinopathy with macular edema, right eye
Diabetes	E11.3212	Type 2 diabetes mellitus with mild non-proliferative diabetic retinopathy with macular edema, left eye
Diabetes	E11.3213	Type 2 diabetes mellitus with mild non-proliferative diabetic retinopathy with macular edema, bilateral
Diabetes	E11.3219	Type 2 diabetes mellitus with mild non-proliferative diabetic retinopathy with macular edema, unspecified eye
Diabetes	E11.329	Type 2 diabetes mellitus with mild non-proliferative diabetic retinopathy without macular edema
Diabetes	E11.3291	Type 2 diabetes mellitus with mild non-proliferative diabetic retinopathy without macular edema, right eye
Diabetes	E11.3292	Type 2 diabetes mellitus with mild non-proliferative diabetic retinopathy without macular edema, left eye
Diabetes	E11.3293	Type 2 diabetes mellitus with mild non-proliferative diabetic retinopathy without macular edema, bilateral
Diabetes	E11.3299	Type 2 diabetes mellitus with mild non-proliferative diabetic retinopathy without macular edema, unspecified eye
Diabetes	E11.331	Type 2 diabetes mellitus with moderate non-proliferative diabetic retinopathy with macular edema
Diabetes	E11.3311	Type 2 diabetes mellitus with moderate non-proliferative diabetic retinopathy with macular edema, right eye
Diabetes	E11.3312	Type 2 diabetes mellitus with moderate non-proliferative diabetic retinopathy with macular edema, left eye
Diabetes	E11.3313	Type 2 diabetes mellitus with moderate non-proliferative diabetic retinopathy with macular edema, bilateral
Diabetes	E11.3319	Type 2 diabetes mellitus with moderate non-proliferative diabetic retinopathy with macular edema, unspecified eye
Diabetes	E11.339	Type 2 diabetes mellitus with moderate non-proliferative diabetic retinopathy without macular edema
Diabetes	E11.3391	Type 2 diabetes mellitus with moderate non-proliferative diabetic retinopathy without macular edema, right eye
Diabetes	E11.3392	Type 2 diabetes mellitus with moderate non-proliferative diabetic retinopathy without macular edema, left eye

© 2025 Gidwani R et al. *JAMA Network Open*.

Condition	Code	Code Descriptions
Diabetes	E11.3393	Type 2 diabetes mellitus with moderate non-proliferative diabetic retinopathy without macular edema, bilateral
Diabetes	E11.3399	Type 2 diabetes mellitus with moderate non-proliferative diabetic retinopathy without macular edema, unspecified eye
Diabetes	E11.341	Type 2 diabetes mellitus with severe non-proliferative diabetic retinopathy with macular edema
Diabetes	E11.3411	Type 2 diabetes mellitus with severe non-proliferative diabetic retinopathy with macular edema, right eye
Diabetes	E11.3412	Type 2 diabetes mellitus with severe non-proliferative diabetic retinopathy with macular edema, left eye
Diabetes	E11.3413	Type 2 diabetes mellitus with severe non-proliferative diabetic retinopathy with macular edema, bilateral
Diabetes	E11.3419	Type 2 diabetes mellitus with severe non-proliferative diabetic retinopathy with macular edema, unspecified eye
Diabetes	E11.349	Type 2 diabetes mellitus with severe non-proliferative diabetic retinopathy without macular edema
Diabetes	E11.3491	Type 2 diabetes mellitus with severe non-proliferative diabetic retinopathy without macular edema, right eye
Diabetes	E11.3492	Type 2 diabetes mellitus with severe non-proliferative diabetic retinopathy without macular edema, left eye
Diabetes	E11.3493	Type 2 diabetes mellitus with severe non-proliferative diabetic retinopathy without macular edema, bilateral
Diabetes	E11.3499	Type 2 diabetes mellitus with severe non-proliferative diabetic retinopathy without macular edema, unspecified eye
Diabetes	E11.351	Type 2 diabetes mellitus with proliferative diabetic retinopathy with macular edema
Diabetes	E11.3511	Type 2 diabetes mellitus with proliferative diabetic retinopathy with macular edema, right eye
Diabetes	E11.3512	Type 2 diabetes mellitus with proliferative diabetic retinopathy with macular edema, left eye
Diabetes	E11.3513	Type 2 diabetes mellitus with proliferative diabetic retinopathy with macular edema, bilateral
Diabetes	E11.3519	Type 2 diabetes mellitus with proliferative diabetic retinopathy with macular edema, unspecified eye
Diabetes	E11.3521	Type 2 diabetes mellitus with proliferative diabetic retinopathy with traction retinal detachment involving the macula, right eye
Diabetes	E11.3522	Type 2 diabetes mellitus with proliferative diabetic retinopathy with traction retinal detachment involving the macula, left eye
Diabetes	E11.3523	Type 2 diabetes mellitus with proliferative diabetic retinopathy with traction retinal detachment involving the macula, bilateral
Diabetes	E11.3529	Type 2 diabetes mellitus with proliferative diabetic retinopathy with traction retinal detachment involving the macula, unspecified eye

© 2025 Gidwani R et al. *JAMA Network Open*.

Condition	Code	Code Descriptions
Diabetes	E11.3531	Type 2 diabetes mellitus with proliferative diabetic retinopathy with traction retinal detachment not involving the macula, right eye
Diabetes	E11.3532	Type 2 diabetes mellitus with proliferative diabetic retinopathy with traction retinal detachment not involving the macula, left eye
Diabetes	E11.3533	Type 2 diabetes mellitus with proliferative diabetic retinopathy with traction retinal detachment not involving the macula, bilateral
Diabetes	E11.3539	Type 2 diabetes mellitus with proliferative diabetic retinopathy with traction retinal detachment not involving the macula, unspecified eye
Diabetes	E11.3541	Type 2 diabetes mellitus with proliferative diabetic retinopathy with combined traction retinal detachment and rhegmatogenous retinal detachment, right eye
Diabetes	E11.3542	Type 2 diabetes mellitus with proliferative diabetic retinopathy with combined traction retinal detachment and rhegmatogenous retinal detachment, left eye
Diabetes	E11.3543	Type 2 diabetes mellitus with proliferative diabetic retinopathy with combined traction retinal detachment and rhegmatogenous retinal detachment, bilateral
Diabetes	E11.3549	Type 2 diabetes mellitus with proliferative diabetic retinopathy with combined traction retinal detachment and rhegmatogenous retinal detachment, unspecified eye
Diabetes	E11.3551	Type 2 diabetes mellitus with stable proliferative diabetic retinopathy, right eye
Diabetes	E11.3552	Type 2 diabetes mellitus with stable proliferative diabetic retinopathy, left eye
Diabetes	E11.3553	Type 2 diabetes mellitus with stable proliferative diabetic retinopathy, bilateral
Diabetes	E11.3559	Type 2 diabetes mellitus with stable proliferative diabetic retinopathy, unspecified eye
Diabetes	E11.359	Type 2 diabetes mellitus with proliferative diabetic retinopathy without macular edema
Diabetes	E11.3591	Type 2 diabetes mellitus with proliferative diabetic retinopathy without macular edema, right eye
Diabetes	E11.3592	Type 2 diabetes mellitus with proliferative diabetic retinopathy without macular edema, left eye
Diabetes	E11.3593	Type 2 diabetes mellitus with proliferative diabetic retinopathy without macular edema, bilateral
Diabetes	E11.3599	Type 2 diabetes mellitus with proliferative diabetic retinopathy without macular edema, unspecified eye
Diabetes	E11.36	Type 2 diabetes mellitus with diabetic cataract
Diabetes	E11.37X1	Type 2 diabetes mellitus with diabetic macular edema, resolved following treatment, right eye
Diabetes	E11.37X2	Type 2 diabetes mellitus with diabetic macular edema, resolved following treatment, left eye

© 2025 Gidwani R et al. *JAMA Network Open*.

Condition	Code	Code Descriptions
Diabetes	E11.37X3	Type 2 diabetes mellitus with diabetic macular edema, resolved following treatment, bilateral
Diabetes	E11.37X9	Type 2 diabetes mellitus with diabetic macular edema, resolved following treatment, unspecified eye
Diabetes	E11.39	Type 2 diabetes mellitus with other diabetic ophthalmic complication
Diabetes	E11.40	Type 2 diabetes mellitus with diabetic neuropathy, unspecified
Diabetes	E11.41	Type 2 diabetes mellitus with diabetic mononeuropathy
Diabetes	E11.42	Type 2 diabetes mellitus with diabetic polyneuropathy
Diabetes	E11.43	Type 2 diabetes mellitus with diabetic autonomic (poly)neuropathy
Diabetes	E11.44	Type 2 diabetes mellitus with diabetic amyotrophy
Diabetes	E11.49	Type 2 diabetes mellitus with other diabetic neurological complication
Diabetes	E11.51	Type 2 diabetes mellitus with diabetic peripheral angiopathy without gangrene
Diabetes	E11.52	Type 2 diabetes mellitus with diabetic peripheral angiopathy with gangrene
Diabetes	E11.59	Type 2 diabetes mellitus with other circulatory complications
Diabetes	E11.610	Type 2 diabetes mellitus with diabetic neuropathic arthropathy
Diabetes	E11.618	Type 2 diabetes mellitus with other diabetic arthropathy
Diabetes	E11.620	Type 2 diabetes mellitus with diabetic dermatitis
Diabetes	E11.621	Type 2 diabetes mellitus with foot ulcer
Diabetes	E11.622	Type 2 diabetes mellitus with other skin ulcer
Diabetes	E11.628	Type 2 diabetes mellitus with other skin complications
Diabetes	E11.630	Type 2 diabetes mellitus with periodontal disease
Diabetes	E11.638	Type 2 diabetes mellitus with other oral complications
Diabetes	E11.641	Type 2 diabetes mellitus with hypoglycemia with coma
Diabetes	E11.649	Type 2 diabetes mellitus with hypoglycemia without coma
Diabetes	E11.65	Type 2 diabetes mellitus with hyperglycemia
Diabetes	E11.69	Type 2 diabetes mellitus with other specified complication
Diabetes	E11.8	Type 2 diabetes mellitus with unspecified complications
Diabetes	E11.9	Type 2 diabetes mellitus without complications
Diabetes	E13.00	Other specified diabetes mellitus with hyperosmolarity without nonketotic hyperglycemic-hyperosmolar coma (NKHHC)
Diabetes	E13.01	Other specified diabetes mellitus with hyperosmolarity with coma
Diabetes	E13.10	Other specified diabetes mellitus with ketoacidosis without coma
Diabetes	E13.11	Other specified diabetes mellitus with ketoacidosis with coma
Diabetes	E13.21	Other specified diabetes mellitus with diabetic nephropathy
Diabetes	E13.22	Other specified diabetes mellitus with diabetic chronic kidney disease

© 2025 Gidwani R et al. *JAMA Network Open*.

Condition	Code	Code Descriptions
Diabetes	E13.29	Other specified diabetes mellitus with other diabetic kidney complication
Diabetes	E13.311	Other specified diabetes mellitus with unspecified diabetic retinopathy with macular edema
Diabetes	E13.319	Other specified diabetes mellitus with unspecified diabetic retinopathy without macular edema
Diabetes	E13.321	Other specified diabetes mellitus with mild non-proliferative diabetic retinopathy with macular edema
Diabetes	E13.3211	Other specified diabetes mellitus with mild non-proliferative diabetic retinopathy with macular edema, right eye
Diabetes	E13.3212	Other specified diabetes mellitus with mild non-proliferative diabetic retinopathy with macular edema, left eye
Diabetes	E13.3213	Other specified diabetes mellitus with mild non-proliferative diabetic retinopathy with macular edema, bilateral
Diabetes	E13.3219	Other specified diabetes mellitus with mild non-proliferative diabetic retinopathy with macular edema, unspecified eye
Diabetes	E13.329	Other specified diabetes mellitus with mild non-proliferative diabetic retinopathy without macular edema
Diabetes	E13.3291	Other specified diabetes mellitus with mild non-proliferative diabetic retinopathy without macular edema, right eye
Diabetes	E13.3292	Other specified diabetes mellitus with mild non-proliferative diabetic retinopathy without macular edema, left eye
Diabetes	E13.3293	Other specified diabetes mellitus with mild non-proliferative diabetic retinopathy without macular edema, bilateral
Diabetes	E13.3299	Other specified diabetes mellitus with mild non-proliferative diabetic retinopathy without macular edema, unspecified eye
Diabetes	E13.331	Other specified diabetes mellitus with moderate non-proliferative diabetic retinopathy with macular edema
Diabetes	E13.3311	Other specified diabetes mellitus with moderate non-proliferative diabetic retinopathy with macular edema, right eye
Diabetes	E13.3312	Other specified diabetes mellitus with moderate non-proliferative diabetic retinopathy with macular edema, left eye
Diabetes	E13.3313	Other specified diabetes mellitus with moderate non-proliferative diabetic retinopathy with macular edema, bilateral
Diabetes	E13.3319	Other specified diabetes mellitus with moderate non-proliferative diabetic retinopathy with macular edema, unspecified eye
Diabetes	E13.339	Other specified diabetes mellitus with moderate non-proliferative diabetic retinopathy without macular edema
Diabetes	E13.3391	Other specified diabetes mellitus with moderate non-proliferative diabetic retinopathy without macular edema, right eye
Diabetes	E13.3392	Other specified diabetes mellitus with moderate non-proliferative diabetic retinopathy without macular edema, left eye

© 2025 Gidwani R et al. *JAMA Network Open*.

Condition	Code	Code Descriptions
Diabetes	E13.3393	Other specified diabetes mellitus with moderate non-proliferative diabetic retinopathy without macular edema, bilateral
Diabetes	E13.3399	Other specified diabetes mellitus with moderate non-proliferative diabetic retinopathy without macular edema, unspecified eye
Diabetes	E13.341	Other specified diabetes mellitus with severe non-proliferative diabetic retinopathy with macular edema
Diabetes	E13.3411	Other specified diabetes mellitus with severe non-proliferative diabetic retinopathy with macular edema, right eye
Diabetes	E13.3412	Other specified diabetes mellitus with severe non-proliferative diabetic retinopathy with macular edema, left eye
Diabetes	E13.3413	Other specified diabetes mellitus with severe non-proliferative diabetic retinopathy with macular edema, bilateral
Diabetes	E13.3419	Other specified diabetes mellitus with severe non-proliferative diabetic retinopathy with macular edema, unspecified eye
Diabetes	E13.349	Other specified diabetes mellitus with severe non-proliferative diabetic retinopathy without macular edema
Diabetes	E13.3491	Other specified diabetes mellitus with severe non-proliferative diabetic retinopathy without macular edema, right eye
Diabetes	E13.3492	Other specified diabetes mellitus with severe non-proliferative diabetic retinopathy without macular edema, left eye
Diabetes	E13.3493	Other specified diabetes mellitus with severe non-proliferative diabetic retinopathy without macular edema, bilateral
Diabetes	E13.3499	Other specified diabetes mellitus with severe non-proliferative diabetic retinopathy without macular edema, unspecified eye
Diabetes	E13.351	Other specified diabetes mellitus with proliferative diabetic retinopathy with macular edema
Diabetes	E13.3511	Other specified diabetes mellitus with proliferative diabetic retinopathy with macular edema, right eye
Diabetes	E13.3512	Other specified diabetes mellitus with proliferative diabetic retinopathy with macular edema, left eye
Diabetes	E13.3513	Other specified diabetes mellitus with proliferative diabetic retinopathy with macular edema, bilateral
Diabetes	E13.3519	Other specified diabetes mellitus with proliferative diabetic retinopathy with macular edema, unspecified eye
Diabetes	E13.3521	Other specified diabetes mellitus with proliferative diabetic retinopathy with traction retinal detachment involving the macula, right eye
Diabetes	E13.3522	Other specified diabetes mellitus with proliferative diabetic retinopathy with traction retinal detachment involving the macula, left eye
Diabetes	E13.3523	Other specified diabetes mellitus with proliferative diabetic retinopathy with traction retinal detachment involving the macula, bilateral
Diabetes	E13.3529	Other specified diabetes mellitus with proliferative diabetic retinopathy with traction retinal detachment involving the macula, unspecified eye

© 2025 Gidwani R et al. *JAMA Network Open*.

Condition	Code	Code Descriptions
Diabetes	E13.3531	Other specified diabetes mellitus with proliferative diabetic retinopathy with traction retinal detachment not involving the macula, right eye
Diabetes	E13.3532	Other specified diabetes mellitus with proliferative diabetic retinopathy with traction retinal detachment not involving the macula, left eye
Diabetes	E13.3533	Other specified diabetes mellitus with proliferative diabetic retinopathy with traction retinal detachment not involving the macula, bilateral
Diabetes	E13.3539	Other specified diabetes mellitus with proliferative diabetic retinopathy with traction retinal detachment not involving the macula, unspecified eye
Diabetes	E13.3541	Other specified diabetes mellitus with proliferative diabetic retinopathy with combined traction retinal detachment and rhegmatogenous retinal detachment, right eye
Diabetes	E13.3542	Other specified diabetes mellitus with proliferative diabetic retinopathy with combined traction retinal detachment and rhegmatogenous retinal detachment, left eye
Diabetes	E13.3543	Other specified diabetes mellitus with proliferative diabetic retinopathy with combined traction retinal detachment and rhegmatogenous retinal detachment, bilateral
Diabetes	E13.3549	Other specified diabetes mellitus with proliferative diabetic retinopathy with combined traction retinal detachment and rhegmatogenous retinal detachment, unspecified eye
Diabetes	E13.3551	Other specified diabetes mellitus with stable proliferative diabetic retinopathy, right eye
Diabetes	E13.3552	Other specified diabetes mellitus with stable proliferative diabetic retinopathy, left eye
Diabetes	E13.3553	Other specified diabetes mellitus with stable proliferative diabetic retinopathy, bilateral
Diabetes	E13.3559	Other specified diabetes mellitus with stable proliferative diabetic retinopathy, unspecified eye
Diabetes	E13.359	Other specified diabetes mellitus with proliferative diabetic retinopathy without macular edema
Diabetes	E13.3591	Other specified diabetes mellitus with proliferative diabetic retinopathy without macular edema, right eye
Diabetes	E13.3592	Other specified diabetes mellitus with proliferative diabetic retinopathy without macular edema, left eye
Diabetes	E13.3593	Other specified diabetes mellitus with proliferative diabetic retinopathy without macular edema, bilateral
Diabetes	E13.3599	Other specified diabetes mellitus with proliferative diabetic retinopathy without macular edema, unspecified eye
Diabetes	E13.36	Other specified diabetes mellitus with diabetic cataract
Diabetes	E13.37X1	Other specified diabetes mellitus with diabetic macular edema, resolved following treatment, right eye
Diabetes	E13.37X2	Other specified diabetes mellitus with diabetic macular edema, resolved following treatment, left eye

© 2025 Gidwani R et al. *JAMA Network Open*.

Condition	Code	Code Descriptions
Diabetes	E13.37X3	Other specified diabetes mellitus with diabetic macular edema, resolved following treatment, bilateral
Diabetes	E13.37X9	Other specified diabetes mellitus with diabetic macular edema, resolved following treatment, unspecified eye
Diabetes	E13.39	Other specified diabetes mellitus with other diabetic ophthalmic complication
Diabetes	E13.40	Other specified diabetes mellitus with diabetic neuropathy, unspecified
Diabetes	E13.41	Other specified diabetes mellitus with diabetic mononeuropathy
Diabetes	E13.42	Other specified diabetes mellitus with diabetic polyneuropathy
Diabetes	E13.43	Other specified diabetes mellitus with diabetic autonomic (poly)neuropathy
Diabetes	E13.44	Other specified diabetes mellitus with diabetic amyotrophy
Diabetes	E13.49	Other specified diabetes mellitus with other diabetic neurological complication
Diabetes	E13.51	Other specified diabetes mellitus with diabetic peripheral angiopathy without gangrene
Diabetes	E13.52	Other specified diabetes mellitus with diabetic peripheral angiopathy with gangrene
Diabetes	E13.59	Other specified diabetes mellitus with other circulatory complications
Diabetes	E13.610	Other specified diabetes mellitus with diabetic neuropathic arthropathy
Diabetes	E13.618	Other specified diabetes mellitus with other diabetic arthropathy
Diabetes	E13.620	Other specified diabetes mellitus with diabetic dermatitis
Diabetes	E13.621	Other specified diabetes mellitus with foot ulcer
Diabetes	E13.622	Other specified diabetes mellitus with other skin ulcer
Diabetes	E13.628	Other specified diabetes mellitus with other skin complications
Diabetes	E13.630	Other specified diabetes mellitus with periodontal disease
Diabetes	E13.638	Other specified diabetes mellitus with other oral complications
Diabetes	E13.641	Other specified diabetes mellitus with hypoglycemia with coma
Diabetes	E13.649	Other specified diabetes mellitus with hypoglycemia without coma
Diabetes	E13.65	Other specified diabetes mellitus with hyperglycemia
Diabetes	E13.69	Other specified diabetes mellitus with other specified complication
Diabetes	E13.8	Other specified diabetes mellitus with unspecified complications
Diabetes	E13.9	Other specified diabetes mellitus without complications
Diabetes	O24.011	Pre-existing type 1 diabetes mellitus, in pregnancy, first trimester
Diabetes	O24.012	Pre-existing type 1 diabetes mellitus, in pregnancy, second trimester
Diabetes	O24.013	Pre-existing type 1 diabetes mellitus, in pregnancy, third trimester

© 2025 Gidwani R et al. *JAMA Network Open*.

Condition	Code	Code Descriptions
Diabetes	O24.019	Pre-existing type 1 diabetes mellitus, in pregnancy, unspecified trimester
Diabetes	O24.02	Pre-existing type 1 diabetes mellitus, in childbirth
Diabetes	O24.03	Pre-existing type 1 diabetes mellitus, in the puerperium
Diabetes	O24.111	Pre-existing type 2 diabetes mellitus, in pregnancy, first trimester
Diabetes	O24.112	Pre-existing type 2 diabetes mellitus, in pregnancy, second trimester
Diabetes	O24.113	Pre-existing type 2 diabetes mellitus, in pregnancy, third trimester
Diabetes	O24.119	Pre-existing type 2 diabetes mellitus, in pregnancy, unspecified trimester
Diabetes	O24.12	Pre-existing type 2 diabetes mellitus, in childbirth
Diabetes	O24.13	Pre-existing type 2 diabetes mellitus, in the puerperium
Diabetes	O24.311	Unspecified pre-existing diabetes mellitus in pregnancy, first trimester
Diabetes	O24.312	Unspecified pre-existing diabetes mellitus in pregnancy, second trimester
Diabetes	O24.313	Unspecified pre-existing diabetes mellitus in pregnancy, third trimester
Diabetes	O24.319	Unspecified pre-existing diabetes mellitus in pregnancy, unspecified trimester
Diabetes	O24.32	Unspecified pre-existing diabetes mellitus in childbirth
Diabetes	O24.33	Unspecified pre-existing diabetes mellitus in the puerperium
Diabetes	O24.811	Other pre-existing diabetes mellitus in pregnancy, first trimester
Diabetes	O24.812	Other pre-existing diabetes mellitus in pregnancy, second trimester
Diabetes	O24.813	Other pre-existing diabetes mellitus in pregnancy, third trimester
Diabetes	O24.819	Other pre-existing diabetes mellitus in pregnancy, unspecified trimester
Diabetes	O24.82	Other pre-existing diabetes mellitus in childbirth
Diabetes	O24.83	Other pre-existing diabetes mellitus in the puerperium
Hypertension	I10	Essential (primary) hypertension.
Hypertension	I11.9	Hypertensive heart disease without heart failure.
Hypertension	I11.0	Hypertensive heart disease with heart failure.
Hypertension	I12.9	Hypertensive chronic kidney disease with stage 1 through stage 4 chronic kidney disease or unspecified chronic kidney disease.
Hypertension	I12.0	Hypertensive chronic kidney disease with stage 5 chronic kidney disease or end-stage renal disease.
Hypertension	I13.0	Hypertensive heart and chronic kidney disease with heart failure and with stage 1 through stage 4 chronic kidney disease, or unspecified chronic kidney disease.
Hypertension	I13.2	Hypertensive heart and chronic kidney disease with heart failure and with stage 5 chronic kidney disease, or end-stage renal disease.

© 2025 Gidwani R et al. *JAMA Network Open*.

Condition	Code	Code Descriptions
Hypertension	I13.10	Hypertensive heart and chronic kidney disease without heart failure and with stage 1 through stage 4 chronic kidney disease, or unspecified chronic kidney disease.
Hypertension	I13.11	Hypertensive heart and chronic kidney disease without heart failure and with stage 5 chronic kidney disease, or end-stage renal disease.
Coronary Artery Disease	I20.0	Unstable angina
Coronary Artery Disease	I20.8	Other forms of angina pectoris
Coronary Artery Disease	I20.9	Angina pectoris, unspecified
Coronary Artery Disease	I24.0	Acute coronary thrombosis not resulting in myocardial infarction
Coronary Artery Disease	I24.1	Dressler's syndrome
Coronary Artery Disease	I24.8	Other forms of acute ischemic heart disease
Coronary Artery Disease	I24.9	Acute ischemic heart disease, unspecified
Coronary Artery Disease	I25.10	Atherosclerotic heart disease of native coronary artery without angina pectoris
Coronary Artery Disease	I25.110	Atherosclerotic heart disease of native coronary artery with unstable angina pectoris
Coronary Artery Disease	I25.111	Atherosclerotic heart disease of native coronary artery with angina pectoris with documented spasm
Coronary Artery Disease	I25.118	Atherosclerotic heart disease of native coronary artery with other forms of angina pectoris
Coronary Artery Disease	I25.119	Atherosclerotic heart disease of native coronary artery with unspecified angina pectoris
Coronary Artery Disease	I25.5	Ischemic cardiomyopathy
Coronary Artery Disease	I25.6	Silent myocardial ischemia
Coronary Artery Disease	I25.700	Atherosclerosis of coronary artery bypass graft(s), unspecified, with unstable angina pectoris
Coronary Artery Disease	I25.701	Atherosclerosis of coronary artery bypass graft(s), unspecified, with angina pectoris with documented spasm
Coronary Artery Disease	I25.708	Atherosclerosis of coronary artery bypass graft(s), unspecified, with other forms of angina pectoris
Coronary Artery Disease	I25.709	Atherosclerosis of coronary artery bypass graft(s), unspecified, with unspecified angina pectoris
Coronary Artery Disease	I25.710	Atherosclerosis of autologous vein coronary artery bypass graft(s) with unstable angina pectoris

© 2025 Gidwani R et al. *JAMA Network Open*.

Condition	Code	Code Descriptions
Coronary Artery Disease	I25.711	Atherosclerosis of autologous vein coronary artery bypass graft(s) with angina pectoris with documented spasm
Coronary Artery Disease	I25.718	Atherosclerosis of autologous vein coronary artery bypass graft(s) with other forms of angina pectoris
Coronary Artery Disease	I25.719	Atherosclerosis of autologous vein coronary artery bypass graft(s) with unspecified angina pectoris
Coronary Artery Disease	I25.720	Atherosclerosis of autologous artery coronary artery bypass graft(s) with unstable angina pectoris
Coronary Artery Disease	I25.721	Atherosclerosis of autologous artery coronary artery bypass graft(s) with angina pectoris with documented spasm
Coronary Artery Disease	I25.728	Atherosclerosis of autologous artery coronary artery bypass graft(s) with other forms of angina pectoris
Coronary Artery Disease	I25.729	Atherosclerosis of autologous artery coronary artery bypass graft(s) with unspecified angina pectoris
Coronary Artery Disease	I25.730	Atherosclerosis of non-autologous biological coronary artery bypass graft(s) with unstable angina pectoris
Coronary Artery Disease	I25.731	Atherosclerosis of non-autologous biological coronary artery bypass graft(s) with angina pectoris with documented spasm
Coronary Artery Disease	I25.738	Atherosclerosis of non-autologous biological coronary artery bypass graft(s) with other forms of angina pectoris
Coronary Artery Disease	I25.739	Atherosclerosis of non-autologous biological coronary artery bypass graft(s) with unspecified angina pectoris
Coronary Artery Disease	I25.750	Atherosclerosis of native coronary artery of transplanted heart with unstable angina
Coronary Artery Disease	I25.751	Atherosclerosis of native coronary artery of transplanted heart with angina pectoris with documented spasm
Coronary Artery Disease	I25.758	Atherosclerosis of native coronary artery of transplanted heart with other forms of angina pectoris
Coronary Artery Disease	I25.759	Atherosclerosis of native coronary artery of transplanted heart with unspecified angina pectoris
Coronary Artery Disease	I25.760	Atherosclerosis of bypass graft of coronary artery of transplanted heart with unstable angina
Coronary Artery Disease	I25.761	Atherosclerosis of bypass graft of coronary artery of transplanted heart with angina pectoris with documented spasm
Coronary Artery Disease	I25.768	Atherosclerosis of bypass graft of coronary artery of transplanted heart with other forms of angina pectoris
Coronary Artery Disease	I25.769	Atherosclerosis of bypass graft of coronary artery of transplanted heart with unspecified angina pectoris
Coronary Artery Disease	I25.790	Atherosclerosis of other coronary artery bypass graft(s) with unstable angina pectoris
Coronary Artery Disease	I25.791	Atherosclerosis of other coronary artery bypass graft(s) with angina pectoris with documented spasm

© 2025 Gidwani R et al. *JAMA Network Open*.

Condition	Code	Code Descriptions
Coronary Artery Disease	I25.798	Atherosclerosis of other coronary artery bypass graft(s) with other forms of angina pectoris
Coronary Artery Disease	I25.799	Atherosclerosis of other coronary artery bypass graft(s) with unspecified angina pectoris
Coronary Artery Disease	I25.810	Atherosclerosis of coronary artery bypass graft(s) without angina pectoris
Coronary Artery Disease	I25.811	Atherosclerosis of native coronary artery of transplanted heart without angina pectoris
Coronary Artery Disease	I25.812	Atherosclerosis of bypass graft of coronary artery of transplanted heart without angina pectoris
Coronary Artery Disease	I25.82	Chronic total occlusion of coronary artery
Coronary Artery Disease	I25.83	Coronary atherosclerosis due to lipid rich plaque
Coronary Artery Disease	I25.84	Coronary atherosclerosis due to calcified coronary lesion
Coronary Artery Disease	I25.89	Other forms of chronic ischemic heart disease
Coronary Artery Disease	I25.9	Chronic ischemic heart disease, unspecified
Coronary Artery Disease	I21.01	ST elevation (STEMI) myocardial infarction involving left main coronary artery
Coronary Artery Disease	I21.02	ST elevation (STEMI) myocardial infarction involving left anterior descending coronary artery
Coronary Artery Disease	I21.09	ST elevation (STEMI) myocardial infarction involving other coronary artery of anterior wall
Coronary Artery Disease	I21.11	ST elevation (STEMI) myocardial infarction involving right coronary artery
Coronary Artery Disease	I21.19	ST elevation (STEMI) myocardial infarction involving other coronary artery of inferior wall
Coronary Artery Disease	I21.21	ST elevation (STEMI) myocardial infarction involving left circumflex coronary artery
Coronary Artery Disease	I21.29	ST elevation (STEMI) myocardial infarction involving other sites
Coronary Artery Disease	I21.3	ST elevation (STEMI) myocardial infarction of unspecified site
Coronary Artery Disease	I21.4	Non-ST elevation (NSTEMI) myocardial infarction
Coronary Artery Disease	I22.0	Subsequent ST elevation (STEMI) myocardial infarction of anterior wall
Coronary Artery Disease	I22.1	Subsequent ST elevation (STEMI) myocardial infarction of inferior wall

© 2025 Gidwani R et al. *JAMA Network Open*.

Condition	Code	Code Descriptions
Coronary Artery Disease	I22.2	Subsequent non-ST elevation (NSTEMI) myocardial infarction
Coronary Artery Disease	I22.8	Subsequent ST elevation (STEMI) myocardial infarction of other sites
Coronary Artery Disease	I22.9	Subsequent ST elevation (STEMI) myocardial infarction of unspecified site
Coronary Artery Disease	I23.0	Hemopericardium as current complication following acute myocardial infarction
Coronary Artery Disease	I23.1	Atrial septal defect as current complication following acute myocardial infarction
Coronary Artery Disease	I23.2	Ventricular septal defect as current complication following acute myocardial infarction
Coronary Artery Disease	I23.3	Rupture of cardiac wall without hemopericardium as current complication following acute myocardial infarction
Coronary Artery Disease	I23.4	Rupture of chordae tendineae as current complication following acute myocardial infarction
Coronary Artery Disease	I23.5	Rupture of papillary muscle as current complication following acute myocardial infarction
Coronary Artery Disease	I23.6	Thrombosis of atrium, auricular appendage, and ventricle as current complications following acute myocardial infarction
Coronary Artery Disease	I23.7	Postinfarction angina
Coronary Artery Disease	I23.8	Other current complications following acute myocardial infarction
Coronary Artery Disease	I25.2	Old myocardial infarction
Coronary Artery Disease	I21.01	ST elevation (STEMI) myocardial infarction involving left main coronary artery
Coronary Artery Disease	I21.02	ST elevation (STEMI) myocardial infarction involving left anterior descending coronary artery
Coronary Artery Disease	I21.09	ST elevation (STEMI) myocardial infarction involving other coronary artery of anterior wall
Coronary Artery Disease	I21.11	ST elevation (STEMI) myocardial infarction involving right coronary artery
Coronary Artery Disease	I21.19	ST elevation (STEMI) myocardial infarction involving other coronary artery of inferior wall
Coronary Artery Disease	I21.21	ST elevation (STEMI) myocardial infarction involving left circumflex coronary artery
Coronary Artery Disease	I21.29	ST elevation (STEMI) myocardial infarction involving other sites
Coronary Artery Disease	I21.3	ST elevation (STEMI) myocardial infarction of unspecified site

© 2025 Gidwani R et al. *JAMA Network Open*.

Condition	Code	Code Descriptions
Coronary Artery Disease	I21.4	Non-ST elevation (NSTEMI) myocardial infarction
Heart Failure	I50.4	Combined systolic (congestive) and diastolic (congestive) heart failure
Heart Failure	I50.40	Unspecified combined systolic (congestive) and diastolic (congestive) heart failure
Heart Failure	I50.42	Chronic combined systolic (congestive) and diastolic (congestive) heart failure
Heart Failure	I50.43	Acute on chronic combined systolic (congestive) and diastolic (congestive) heart failure
Heart Failure	I50.3	Diastolic (congestive) heart failure
Heart Failure	I50.30	Unspecified diastolic (congestive) heart failure
Heart Failure	I50.32	Chronic diastolic (congestive) heart failure
Heart Failure	I50.33	Acute on chronic diastolic (congestive) heart failure
Heart Failure	I50.81	Right heart failure
Heart Failure	I50.81	Right heart failure, unspecified
Heart Failure	I50.812	Chronic right heart failure
Heart Failure	I50.813	Acute on chronic right heart failure
Heart Failure	I50.2	Systolic (congestive) heart failure
Heart Failure	I50.20	Unspecified systolic (congestive) heart failure
Heart Failure	I50.22	Chronic systolic (congestive) heart failure
Heart Failure	I50.23	Acute on chronic systolic (congestive) heart failure
Heart Failure	I50	Heart Failure
Heart Failure	I50.I	Left ventricular failure, unspecified
Heart Failure	I50.8	Other heart failure
Heart Failure	I50.814	Right heart failure due to left heart failure
Heart Failure	I50.82	Biventricular heart failure
Heart Failure	I50.83	High output heart failure
Heart Failure	I50.84	End stage heart failure
Heart Failure	I50.89	Other heart failure
Heart Failure	I50.9	Heart failure, unspecified
Asthma	J45.20	Mild intermittent asthma, uncomplicated
Asthma	J45.21	Mild intermittent asthma with (acute) exacerbation
Asthma	J45.22	Mild intermittent asthma with status asthmaticus
Asthma	J45.30	Mild persistent asthma, uncomplicated
Asthma	J45.31	Mild persistent asthma with (acute) exacerbation
Asthma	J45.32	Mild persistent asthma with status asthmaticus
Asthma	J45.40	Moderate persistent asthma, uncomplicated

© 2025 Gidwani R et al. *JAMA Network Open*.

Condition	Code	Code Descriptions
Asthma	J45.41	Moderate persistent asthma with (acute) exacerbation
Asthma	J45.42	Moderate persistent asthma with status asthmaticus
Asthma	J45.50	Severe persistent asthma, uncomplicated
Asthma	J45.51	Severe persistent asthma with (acute) exacerbation
Asthma	J45.52	Severe persistent asthma with status asthmaticus
Asthma	J45.901	Unspecified asthma with (acute) exacerbation
Asthma	J45.902	Unspecified asthma with status asthmaticus
Asthma	J45.909	Unspecified asthma, uncomplicated
Asthma	J45.990	Exercise induced bronchospasm
Asthma	J45.991	Cough variant asthma
Asthma	J45.998	Other asthma
Major Depressive Disorder	F32	Major depressive disorder, single episode
Major Depressive Disorder	F32.0	Major depressive disorder, single episode, mild
Major Depressive Disorder	F32.1	Major depressive disorder, single episode, moderate
Major Depressive Disorder	F32.2	Major depressive disorder, single episode, severe without psychotic features
Major Depressive Disorder	F32.3	Major depressive disorder, single episode, severe with psychotic features
Major Depressive Disorder	F32.4	Major depressive disorder, single episode, in partial remission
Major Depressive Disorder	F33	Major depressive disorder, recurrent
Major Depressive Disorder	F33.0	Major depressive disorder, recurrent, mild
Major Depressive Disorder	F33.1	Major depressive disorder, recurrent, moderate
Major Depressive Disorder	F33.2	Major depressive disorder, recurrent severe without psychotic features
Major Depressive Disorder	F33.3	Major depressive disorder, recurrent, severe with psychotic symptoms
Major Depressive Disorder	F33.4	Major depressive disorder, recurrent, in remission
Major Depressive Disorder	F33.41	Major depressive disorder, recurrent, in partial remission
Major Depressive Disorder	F33.9	Major depressive disorder, recurrent, unspecified
Major Depressive Disorder	F32.0	Major depressive disorder, single episode, mild

© 2025 Gidwani R et al. *JAMA Network Open*.

Condition	Code	Code Descriptions
Major Depressive Disorder	F32.1	Major depressive disorder, single episode, moderate
Major Depressive Disorder	F32.2	Major depressive disorder, single episode, severe without psychotic features
Major Depressive Disorder	F32.3	Major depressive disorder, single episode, severe with psychotic features
Major Depressive Disorder	F32.4	Major depressive disorder, single episode, in partial remission
Major Depressive Disorder	F33.0	Major depressive disorder, recurrent, mild
Major Depressive Disorder	F33.1	Major depressive disorder, recurrent, moderate
Major Depressive Disorder	F33.2	Major depressive disorder, recurrent severe without psychotic features
Major Depressive Disorder	F33.3	Major depressive disorder, recurrent, severe with psychotic symptoms
Major Depressive Disorder	F33.41	Major depressive disorder, recurrent, in partial remission
Major Depressive Disorder	F33.9	Major depressive disorder, recurrent, unspecified

© 2025 Gidwani R et al. *JAMA Network Open*.

Table A2: Recommended Medical Care: Two Conditions

Conditions	Recommended Clinical Care Utilization
Asthma and Diabetes<br>(no retinopathy)	2 visits a year with a PCP OR 2 visits a year with a pulmonologist *AND* 1 with an endocrinologist OR 1 visit per year with PCP *AND* 1 visit per year with a pulmonologist
Asthma and Diabetes<br>(with retinopathy)	2 visits a year with a PCP AND an eye exam; or<br>2 visits a year with a pulmonologist AND 1 visit with an endocrinologist AND an eye exam; or<br>1 visit per year with a PCP AND 1 visit per year with a pulmonologist AND an eye exam
Asthma and Heart Failure	2 visits per year with a PCP; or<br>2 visits per year with a pulmonologist AND 1 visit per year with a cardiologist; or<br>2 visits per year with a pulmonologist AND 1 visit per year with a PCP
Asthma and Major Depressive Disorder	*If the person is not on medication for Major Depressive Disorder (MDD):*<br>2 visits a year with a PCP where there is a diagnosis of Major Depressive Disorder or a psychotherapy CPT code on at least one claim<br><br>*If the person is on medication for Major Depressive Disorder (MDD):*<br>2 visits a year with a PCP that has a Major Depressive Disorder diagnosis on both visits; or<br>2 visits with a psych professional and 2 visits with a pulmonologist; or<br>1 visit year with PCP with Major Depressive Disorder dx or psychotherapy CPT code on the visit, 1 visit a year with a pulmonologist, and 1 visit a year with a psych prof; or<br>2 visits a year with PCP (no diagnosis or CPT codes required) and 2 visits a year with a psych professional
Coronary Artery Disease and Asthma	2 visits per year with a PCP; or<br>2 visits per year with a pulmonologist AND 1 visit per year with a cardiologist; or<br>2 visits per year with a pulmonologist AND 1 visit per year with a PCP
Coronary Artery Disease and Diabetes	1 visit a year with a PCP; or<br>1 visit a year with a PCP AND 1 with a cardiologist; or<br>1 visit a year with a PCP and an endocrinologist
Coronary Artery Disease and Heart Failure	1 visit per year with a PCP or cardiologist
Coronary Artery Disease and Major Depressive Disorder	*If the person is not on medication for Major Depressive Disorder (MDD):*<br>1 visit a year with a PCP with Major Depressive Disorder or a psychotherapy CPT code on the claim; or<br>1 visit a year with a psych professional AND 1 visit a year with a cardiologist or a PCP with no diagnosis necessary on the claim.

© 2025 Gidwani R et al. *JAMA Network Open*.

Conditions	Recommended Clinical Care Utilization
	If the person is on medication for Major Depressive Disorder (MDD):<br>2 visits a year with a PCP that has a Major Depressive Disorder diagnosis or psychotherapy CPT code on both visits; or<br>2 visits with a psych prof and 1 visit with a cardiologist; or<br>1 visit a year with PCP with Major Depressive Disorder dx AND 1 visit a year with a psych prof; or<br>1 visit a year with PCP (no diagnosis or CPT code required) and 2 visits a year with a psych professional.
Diabetes (with retinopathy) and Heart Failure	1 visit a year with a PCP AND an eye exam; or<br>1 visit a year with an endocrinologist AND 1 visit a year with a cardiologist AND an eye exam; or<br>1 visit a year with an endocrinologist + 1 visit a year with a PCP AND an eye exam; or<br>1 visit a year with a cardiologist + 1 visit a year with a PCP AND an eye exam
Diabetes and Heart Failure	1 visit a year with a PCP; or<br>1 visit a year with an endocrinologist AND 1 visit a year with a cardiologist; or<br>1 visit a year with a endocrinologist + 1 visit a year with a PCP; or<br>1 visit a year with a cardiologist + 1 visit a year with a PCP
Diabetes and Hypertension	1 visit a year with a PCP; or<br>1 visit a year with an endocrinologist AND 1 visit a year with a cardiologist; or<br>1 visit a year with an endocrinologist + 1 visit a year with a PCP; or<br>1 visit a year with a cardiologist + 1 visit a year with a PCP
Diabetes with retinopathy and Hypertension	1 visit a year with a PCP and one eye exam; or<br>1 visit a year with an endocrinologist AND 1 visit a year with a cardiologist AND an eye exam; or<br>1 visit a year with an endocrinologist + 1 visit a year with a PCP AND an eye exam; or<br>1 visit a year with a cardiologist + 1 visit a year with a PCP AND an eye exam
Heart Failure and Hypertension	1 visit per year with a PCP or cardiologist
Heart Failure and Major Depressive Disorder	*If the person is not on medication for Major Depressive Disorder (MDD)::*<br>1 visit a year with a PCP with a diagnosis Major Depressive Disorder on the claim or a psychotherapy CPT; or<br>1 visit a year with a psych professional AND 1 visit a year with a cardiologist or a PCP with no diagnosis necessary on the claim.<br><br>*If the person is on medication for Major Depressive Disorder (MDD):*<br>2 visits a year with a PCP that has a Major Depressive Disorder diagnosis or psychotherapy CPT on both visits; or

© 2025 Gidwani R et al. *JAMA Network Open*.

**Conditions**	**Recommended Clinical Care Utilization**
	2 visits with a psych prof and 1 visit with a cardiologist; or<br>1 visit year with PCP with Major Depressive Disorder dx or psychotherapy CPT AND 1 visit a year with a psych prof; or<br>1 visit a year with PCP (no diagnosis or CPT code required) and 2 visits a year with a psych professional.
Hypertension and Asthma	2 visits per year with a PCP; or<br>2 visits per year with a pulmonologist AND 1 visit per year with a cardiologist; or<br>2 visits per year with a pulmonologist AND 1 visit per year with a PCP
Hypertension and Coronary Artery Disease	1 visit per year with a PCP or cardiologist
Hypertension and Major Depressive Disorder	*If the person is not on medication for Major Depressive Disorder (MDD):*<br>1 visit a year with a PCP, with a diagnosis of Major Depressive Disorder or a psychotherapy CPT code on the claim; or<br>1 visit a year with a psych professional AND 1 visit a year with a cardiologist or a PCP with no diagnosis necessary on the claim.<br><br>*If the person is on medication for Major Depressive Disorder (MDD):*<br>2 visits a year with a PCP that has a Major Depressive Disorder diagnosis or a psychotherapy CPT code on both visits; or<br>2 visits with a psych prof and 1 visit with a cardiologist; or<br>1 visit a year with PCP with Major Depressive Disorder dx or psychotherapy CPT code on the claim AND 1 visit a year with a psych prof; or<br>1 visit a year with PCP (no diagnosis or CPT code required) and 2 visits a year with a psych professional.
Major Depressive Disorder and Diabetes (no retinopathy)	*If the person is not on medication for Major Depressive Disorder (MDD):*<br>1 visit a year with a PCP, with a diagnosis of Major Depressive Disorder or a psychotherapy CPT on the claim; or<br>1 visit a year with a psych professional AND 1 visit a year with an endocrinologist or a PCP with no diagnosis or CPT necessary on the claim<br><br>*If the person is on medication for Major Depressive Disorder (MDD):*: a) 2 visits a year with a PCP that has a Major Depressive Disorder diagnosis or psychotherapy CPT on both visits; OR b) 2 visits with a psych prof and 1 visit with a endocrinologist; OR c) 1 visit year with PCP with Major Depressive

© 2025 Gidwani R et al. *JAMA Network Open*.

**Conditions**	**Recommended Clinical Care Utilization**
	Disorder dx and psychotherapy CPT AND 1 visit a year with a psych prof; d) 1 visit a year with PCP (no dx required) and 2 visits a year with a psych professional.
Major Depressive Disorder and Diabetes (with retinopathy)	*If the person is not on medication for Major Depressive Disorder (MDD):*<br>1 visit a year with a PCP with a diagnosis of Major Depressive Disorder on the claim or a psychotherapy CPT code AND an eye exam; or<br>1 visit a year with a psych professional AND (1 visit a year with an endocrinologist or a PCP (with no diagnosis or CPT code necessary on the claim) AND an eye exam<br><br>*If the person is on medication for Major Depressive Disorder (MDD):*<br>2 visits a year with a PCP that has a Major Depressive Disorder diagnosis or psychotherapy CPT code on both visits AND an eye exam; or<br>2 visits with a psych prof and 1 visit with an endocrinologist AND an eye exam; or<br>1 visit year with PCP with Major Depressive Disorder diagnosis or CPT code for psychotherapy on the claim AND 1 visit a year with a psych prof AND an eye exam; or<br>d) 1 visit a year with PCP (no diagnosis or CPT code required) and 2 visits a year with a psych professional AND an eye exam
**Conditions**	**Recommended Lab Utilization (Annual)**
Diabetes and Hypertension	If the patient is on an ACE, ARB, or diuretic – one potassium/serum creatinine lab, 2 HbA1C tests 90 days apart, eGFr test, urine albumin test
Diabetes and Hypertension	If the patient is not on an ACE, ARB, or diuretic -- basic serum creatinine lab, 2 HbA1C tests 90 days apart, eGFr test, urine albumin test
**Conditions**	**Recommended Prescription Drug Utilization**
Diabetes and Hypertension	>= 80% of days covered by ACE or ARBs
Heart Failure and Hypertension	>=80% of days covered by ACE or ARBs and beta-blockers for patients who have reduced ejection fraction
Coronary Artery Disease and Diabetes	>=80% of days covered by statins, beta blocker and ACE or ARBs
Coronary Artery Disease and Asthma	>=80% of days covered by statins, beta blockers and if on LABAs, 80% of those days are covered by corticosteroids
Asthma and Diabetes	>=80% of days covered by ACE or ARBs and if on LABAs, 80% of those days are covered by corticosteroids
Asthma and Hypertension	if on LABAs, >= 80% of those days are covered by corticosteroids

© 2025 Gidwani R et al. *JAMA Network Open*.

Conditions	Recommended Clinical Care Utilization
Coronary Artery Disease and Hypertension	>=80% of days covered by statins, beta blockers.
Heart Failure and Coronary Artery Disease	If reduced ejection fraction, then >=80% of days covered by statins, beta blockers, and ACEs or ARBs. If no reduced ejection fraction, >=80% of days covered by statins and beta blockers
Heart Failure and Diabetes	If ejection fraction, then >=80% of days covered by statins, beta blockers, and ACEs or ARBs. If no educed ejection fraction, then >=80% of days covered by ACE or ARBs
Asthma and Heart Failure	If reduced ejection fraction, then >=80% of days covered by statins and beta blockers, AND if on LABAs, >=80% of those days are covered by corticosteroids. If no reduced ejection fraction AND if on LABAs, >=80% of those days are covered by corticosteroids.
Major Depressive Disorder and Asthma	If on prescription drugs for MDD, >=80% of days covered by antidepressants, AND if on LABAs, >=80% of those days are covered by corticosteroids.
Major Depressive Disorder and Diabetes	If on prescription drugs for MDD, >=80% of days covered by antidepressants, AND >=80% of days covered by an ACE or ARB (note we assume that people with diabetes have hypertension as well)
Major Depressive Disorder and Hypertension	If on prescription drugs for MDD, >=80% of days covered by antidepressants
Major Depressive Disorder and Coronary Artery Disease	If on prescription drugs for MDD, >=80% of days covered by antidepressants and >=80% of days covered by statins, beta blockers.
Major Depressive Disorder and Heart Failure	If on prescription drugs for MDD, >=80% of days covered by antidepressants. If reduced ejection fraction, >=80% of days covered by an ACE or ARB AND a beta blocker.
Major Depressive Disorder and Asthma	If not on prescription drugs for MDD, and if on LABAs, >=80% of days covered by ICS
Major Depressive Disorder and Diabetes	If not on prescription drugs for MDD, >= 80% of days covered by ACE or ARBs
Major Depressive Disorder and Coronary Artery Disease	If not on prescription drugs for MDD, >= 80% of days covered by statins and beta blockers
Major Depressive Disorder and Heart Failure	If not on prescription drugs for MDD, and reduced ejection fraction, >=80% of days covered with statins, beta blockers. If no reduced ejection fraction, no drugs required

© 2025 Gidwani R et al. *JAMA Network Open*.

Table A3: CPT Codes Used to Assess Office Visits for Chronic Illness Management

CPT Code	Description
90832	Psychotherapy, 30 minutes with patient
90833	Psychotherapy, 30 minutes with patient when performed with an evaluation and management service (List separately in addition to the code for primary procedure)
90834	Psychotherapy, 45 minutes with patient
90836	Psychotherapy, 45 minutes with patient when performed with an evaluation and management service (List separately in addition to the code for primary procedure)
90837	Psychotherapy, 60 minutes with patient
90838	Psychotherapy, 60 minutes with patient when performed with an evaluation and management service (List separately in addition to the code for primary procedure)
90845	Psychoanalysis
90847	Family psychotherapy (conjoint psychotherapy) (with patient present), 50 minutes
90853	Group psychotherapy (other than of a multiple-family group)
90863	Pharmacologic management, including prescription and review of medication, when performed with psychotherapy services (list separately in addition to the code for primary procedure)
90839	Psychotherapy for crisis; first 60 minutes*Billed for the first 60 minutes of psychotherapy for a patient in crisis, and add-on code 90840 billed for each additional 30 minutes
90840	Psychotherapy for crisis; each additional 30 minutes (list separately in addition to code for primary service)
90875	Interactive complexity (List separately in addition to the code for primary procedure)Can be reported with appropriate primary procedure (90791, 90832, 90834, 90837, or 90853) if at least one of four specific complicating factor(s) are present.

99202	Office or other outpatient visit for the evaluation and management of a new patient, which requires a medically appropriate history and/or examination and straightforward medical decision making. When using time for code selection, 15-29 minutes of total time is spent on the date of the encounter).
99203	Office or other outpatient visit for the evaluation and management of a new patient, which requires a medically appropriate history and/or examination and low level of medical decision making. When using time for code selection, 30-44 minutes of total time is spent on the date of the encounter.
99204	Office or other outpatient visit for the evaluation and management of a new patient, which requires a medically appropriate history and/or examination and moderate level of medical decision making. When using time for code selection, 45-59 minutes of total time is spent on the date of the encounter.
99205	Office or other outpatient visit for the evaluation and management of a new patient, which requires a medically appropriate history and/or examination and high level of medical decision making. When using time for code selection, 60-74 minutes of total time is spent on the date of the encounter.
99211	Office or other outpatient visit for the evaluation and management of an established patient, that may not require the presence of a physician or other qualified health care professional. Usually, the presenting problem(s) are minimal.
99212	Office or other outpatient visit for the evaluation and management of an established patient, which requires a medically appropriate history and/or examination and straightforward medical decision making. When using time for code selection, 10-19 minutes of total time is spent on the date of the encounter.
99213	Office or other outpatient visit for the evaluation and management of an established patient, which requires a medically appropriate history and/or examination and low level of medical decision making. When using time for code selection, 20-29 minutes of total time is spent on the date of the encounter.

99214	Office or other outpatient visit for the evaluation and management of an established patient, which requires a medically appropriate history and/or examination and moderate level of medical decision making. When using time for code selection, 30-39 minutes of total time is spent on the date of the encounter.
99215	Office or other outpatient visit for the evaluation and management of an established patient, which requires a medically appropriate history and/or examination and high level of medical decision making. When using time for code selection, 40-54 minutes of total time is spent on the date of the encounter.

Table A4: Full Model Results, Recommended Medical Care

		Clinical Care	Prescription Drugs	Labs	Composite Measure
HDHP*post±		-0.031***	-0.090***	-0.057***	-0.047***
	95% CI	(-0.049 - -0.012)	(-0.118 - -0.062)	(-0.082 - -0.032)	(-0.062 - -0.033)
	p-value	0.001	0.000	0.000	0.000
HDHP±		0.093***	0.052***	0.034***	0.061***
	95% CI	(0.083 - 0.104)	(0.034 - 0.069)	(0.020 - 0.048)	(0.053 - 0.069)
	p-value	0.000	0.000	0.000	0.000
post		-0.025***	0.008***	0.005***	-0.010***
	95% CI	(-0.027 - -0.023)	(0.005 - 0.011)	(0.003 - 0.008)	(-0.012 - -0.009)
	p-value	0.000	0.000	0.000	0.000
Coronary Artery Disease		0.107***	-0.427***	0.020*	-0.095***
	95% CI	(0.094 - 0.120)	(-0.454 - -0.399)	(-0.004 - 0.043)	(-0.107 - -0.083)
	p-value	0.000	0.000	0.100	0.000
Hypertension		0.077***	0.025***	0.017**	0.073***
	95% CI	(0.066 - 0.087)	(0.008 - 0.042)	(0.001 - 0.032)	(0.065 - 0.081)

	p-value	0.000	0.004	0.038	0.000
Heart Failure		0.102***	-0.330***	0.145***	0.020
	95% CI	(0.064 - 0.140)	(-0.405 - -0.254)	(0.098 - 0.192)	(-0.014 - 0.055)
	p-value	0.000	0.000	0.000	0.246
Diabetes		0.026***	-0.192***	-0.381***	-0.160***
	95% CI	(0.017 - 0.035)	(-0.214 - -0.170)	(-0.393 - -0.369)	(-0.167 - -0.153)
	p-value	0.000	0.000	0.000	0.000
Asthma		-0.126***	-0.663***	0.039***	-0.312***
	95% CI	(-0.142 - -0.111)	(-0.688 - -0.637)	(0.015 - 0.062)	(-0.322 - -0.302)
	p-value	0.000	0.000	0.001	0.000
Major Depressive Disorder		-0.158***	-0.109***	0.039***	-0.104***
	95% CI	(-0.175 - -0.142)	(-0.135 - -0.084)	(0.010 - 0.068)	(-0.117 - -0.091)
	p-value	0.000	0.000	0.009	0.000
Family (vs) individual insurance plan		0.037***	0.009	0.009	0.019***
	95% CI	(0.028 - 0.045)	(-0.007 - 0.024)	(-0.002 - 0.020)	(0.012 - 0.026)
	p-value	0.000	0.272	0.127	0.000
Age 18-34		-0.117***	-0.093***	-0.071***	-0.112***
	95% CI	(-0.134 - -0.100)	(-0.113 - -0.074)	(-0.096 - -0.047)	(-0.124 - -0.099)
	p-value	0.000	0.000	0.000	0.000
Age 35-44		-0.040***	-0.040***	-0.046***	-0.044***
	95% CI	(-0.053 - -0.027)	(-0.059 - -0.020)	(-0.063 - -0.029)	(-0.054 - -0.033)
	p-value	0.000	0.000	0.000	0.000
Age 55-64		0.027***	0.037***	0.031***	0.029***
	95% CI	(0.019 - 0.035)	(0.021 - 0.052)	(0.021 - 0.041)	(0.022 - 0.035)
	p-value	0.000	0.000	0.000	0.000
Northeast Region		0.081***	0.052***	0.030***	0.058***
	95% CI	(0.069 - 0.093)	(0.033 - 0.071)	(0.014 - 0.045)	(0.048 - 0.067)
	p-value	0.000	0.000	0.000	0.000
North Region		0.063***	0.040	-0.007	0.043***
	95% CI	(0.019 - 0.107)	(-0.040 - 0.121)	(-0.054 - 0.040)	(0.010 - 0.075)

	p-value	0.005	0.326	0.774	0.009
South Region		0.077***	-0.013*	0.014**	0.041***
	95% CI	(0.067 - 0.086)	(-0.029 - 0.002)	(0.002 - 0.026)	(0.033 - 0.048)
	p-value	0.000	0.098	0.022	0.000
Unknown region		-0.066	-0.328***	-0.102**	-0.108
	95% CI	(-0.201 - 0.069)	(-0.570 - -0.086)	(-0.193 - -0.011)	(-0.246 - 0.029)
	p-value	0.337	0.008	0.029	0.123
Female		0.013***	-0.024***	-0.007	0.002
	95% CI	(0.006 - 0.020)	(-0.037 - -0.012)	(-0.017 - 0.002)	(-0.004 - 0.008)
	p-value	0.000	0.000	0.122	0.523
Constant		0.585***	0.698***	0.615***	0.581***
		(0.568 - 0.601)	(0.664 - 0.732)	(0.593 - 0.638)	(0.568 - 0.594)
Observations		343,137	126,617	288,745	343,137

±HDHP is predicted by (instrumented by) presence in a restricted-choice firm.
Reference category for region is West. Reference category for age is 45-54.
Models combine instrumental variables with difference-in-difference models and employ entropy balancing weights. Regression coefficients represent percentage point changes.

Table A5. Model Results, Operationalized Using Instrumented Difference-in-difference Models with Entropy Balancing Weights, excluding 4 care recommendations with Grade C or E evidence*±

Models	Clinic Visits	Prescription Drugs	Labs	Composite Measure
**First Stage**	0.520***	0.512***	0.508***	0.520***
95% CI	(0.510 - 0.531)	(0.495 - 0.529)	(0.496 - 0.519)	(0.509 - 0.530)
p-value	<0.001	<0.001	<0.001	<0.001
F-Statistic	9,394.64	3,477.19	7,462.94	9,511.85
**Reduced Form**	-0.016***	-0.046***	-0.026***	-0.023***
95% CI	(-0.026 - -0.006)	(-0.060 - -0.031)	(-0.039 - -0.013)	(-0.031 - -0.016)
p-value	0.001	0.000	0.000	0.000

**Second Stage**	**-0.030*** **	**-0.090*** **	**-0.051*** **	**-0.045*** **
95% CI	(-0.049 - -0.012)	(-0.118 - -0.062)	(-0.076 - -0.026)	(-0.059 - -0.031)
p-value	0.001	0.000	0.000	0.000
**n**	338,169	126,617	288,745	343,137
**Treatment**	8,826	3,452	7,369	8,969
**Control**	329,343	123,165	281,376	334,168
**Predicted probability of receiving care****				
**HDHP**	71.9%	30.9%	52.4%	57.7%
95% CI	70.5% to 73.4%	28.7% to 33.0%	50.5% to 54.4%	56.6% to 58.8%
**non-HDHP**	75.0%	39.8%	57.5%	62.2%
95% CI	74.4% to 75.6%	38.9% to 40.8%	56.7% to 58.3%	61.7% to 62.7%

* The first stage models use restricted-choice firm interacted with the time variable (post) to predict the effect of a firm who switched to restricted choice precipitating an individual's new enrollment into a HDHP. F-statistics differ across clinic visit, prescription drug and lab outcomes as the sample size for each of these outcomes varies (e.g., all persons in the cohort were eligible for a clinic visit, but not all persons in the cohort are eligible for prescription drugs).

The reduced form models use presence in restricted-choice firm (RCF) to evaluate outcomes of care. The second stage regresses the outcome of recommended medical care on the predicted value of individual HDHP enrollment, which was obtained from the first stage. The main beta coefficient of interest comes from the second stage models, which use an individual's predicted new enrollment into a HDHP (based on presence in a restricted-choice firm) to evaluate outcomes of care; these beta-coefficients represent the percentage point change in the outcome associated with new enrollment in a HDHP.

** Probabilities of received care are predicted from the IV-DiD models and represent the absolute likelihood of receiving care if in an HDHP. All $p < 0.05$

Table A6. Main Results from Sensitivity Analyses, Models 1a-1e ±

Model Type					
**Model 1a. DiD, Treatment Group is Persons Enrolled in a HDHP_HSA plan**		**Clinic Visits Cohort**	**Prescription Drug Cohort**	**Lab Cohort**	**Composite Measure Cohort**
	**DiD**	**-0.014****	**-0.059*****	**-0.036*****	**-0.028*****
	95% CI	(-0.027 - -0.001)	(-0.077 - -0.040)	(-0.052 - -0.020)	(-0.037 - -0.018)
	p-value	0.03	<0.001	<0.001	<0.001
	n	323,282	119,933	272,217	323,282
	Treatment	5,911	2,245	4,641	5,911
	Control	317,371	117,688	267,576	317,371
**Model 1b. IV-DiD. Treatment Group is Persons Enrolled in a HDHP_HSA plan, as instrumented by presence in RCF**		**Clinic Visits Cohort**	**Prescription Drug Cohort**	**Lab Cohort**	**Composite Measure Cohort**
	**First Stage**	0.996***	0.996***	0.997***	0.996***
	95% CI	(0.996 - 0.996)	(0.995 - 0.997)	(0.996 - 0.997)	(0.996 - 0.996)
	p-value	<0.001	<0.001	<0.001	<0.001
	**Reduced Form**	-0.024***	-0.065***	-0.033***	-0.032***
	95% CI	(-0.036 - -0.011)	(-0.084 - -0.046)	(-0.049 - -0.017)	(-0.042 - -0.023)
	p-value	<0.001	<0.001	<0.001	<0.001
	**Second Stage**	**-0.024*****	**-0.065*****	**-0.034*****	**-0.032*****
	95% CI	(-0.036 - -0.011)	(-0.084 - -0.046)	(-0.050 - -0.018)	(-0.042 - -0.023)

		Clinic Visits Cohort	Prescription Drug Cohort	Lab Cohort	Composite Measure Cohort
	p-value	<0.001	<0.001	<0.001	<0.001
	n	340,014	125,387	286,036	340,014
	Treatment	5,846	2,221	4,660	5,846
	Control	334,168	123,166	281,376	334,168
**Model 1c. IV-DiD. Treatment Group is Persons Enrolled in a HDHP_HSA plan, as instrumented by presence in RCF. Persons who were enrolled in a HDHP in the pre-period were dropped from the cohort.**		**Clinic Visits Cohort**	**Prescription Drug Cohort**	**Lab Cohort**	**Composite Measure Cohort**
	**First Stage**	0.673***	0.660***	0.654***	0.673***
	95% CI	(0.662 - 0.684)	(0.642 - 0.678)	(0.642 - 0.666)	(0.662 - 0.684)
	p-value	<0.001	<0.001	<0.001	<0.001
	**Reduced Form**	-0.020***	-0.056***	-0.038***	-0.031***
	95% CI	(-0.031 - -0.009)	(-0.073 - -0.040)	(-0.052 - -0.023)	(-0.040 - -0.023)
	p-value	<0.001	<0.001	<0.001	<0.001
	**Second Stage**	-0.030***	-0.085***	-0.058***	-0.046***
	95% CI	(-0.046 - -0.013)	(-0.111 - -0.060)	(-0.080 - -0.036)	(-0.059 - -0.034)
	p-value	<0.001	<0.001	<0.001	<0.001
	n	324,671	120,380	273,392	324,671
	Treatment	6,910	2,680	5,698	6,910
	Control	317,761	117,700	267,694	317,761
**Model 1d. IV-DiD. Clustered observations**	**Models**	**Clinic Visits Cohort**	**Prescription Drug Cohort**	**Lab Cohort**	**Composite Measure Cohort**
	**First Stage**	0.520***	0.512***	0.508***	0.520***

**within "proxy" Firm ID**$^{\Psi}$	95% CI	(0.516 - 0.524)	(0.309 - 0.714)	(0.330 - 0.686)	(0.329 - 0.710)
	p-value	< 0.001	< 0.001	< 0.001	< 0.001
	**Reduced Form**	-0.016	-0.046***	-0.029***	-0.025***
	95% CI	(-0.042 - 0.010)	(-0.052 - -0.039)	(-0.034 - -0.024)	(-0.038 - -0.012)
	p-value	0.21	< 0.001	< 0.001	< 0.001
	**Second Stage**	**-0.031**	**-0.090*** **	**-0.057*** **	**-0.047*** **
	95% CI	(-0.079 - 0.017)	(-0.102 - -0.078)	(-0.067 - -0.047)	(-0.071 - -0.024)
	p-value	0.207	< 0.001	< 0.001	< 0.001
	n	343,137	126,617	288,745	343,137
	Treatment	8,969	3,452	7,369	8,969
	Control	334,168	123,165	281,376	334,168
**Model 1e. IV-DiD. Uses 2016-2019 data**	**Models**	**Clinic Visits Cohort**	**Prescription Drug Cohort**	**Lab Cohort**	**Composite Measure Cohort**
	**First Stage**	0.504***	0.498***	0.494***	0.504***
	95% CI	(0.492 - 0.517)	(0.479 - 0.518)	(0.480 - 0.507)	(0.492 - 0.517)
	p-value	< 0.001	< 0.001	< 0.001	< 0.001
	**Reduced Form**	-0.019***	-0.039***	-0.019***	-0.022***
	95% CI	(-0.029 - -0.010)	(-0.053 - -0.024)	(-0.031 - -0.006)	(-0.029 - -0.014)
	p-value	< 0.001	< 0.001	< 0.001	< 0.001

	**Second Stage**	**-0.038*** **	**-0.078*** **	**-0.038*** **	**-0.043*** **
	95% CI	(-0.058 - -0.019)	(-0.106 - -0.049)	(-0.063 - -0.013)	(-0.058 - -0.028)
	p-value	0	0	0.003	0
	n	230,807	84,969	193,073	230,807
	Treatment	7,377	2,824	6,041	7,377
	Control	223,430	82,145	187,032	223,430

± IV = instrumental variable; DiD = difference-in-differences; RCF = restricted-choice firm. Beta-coefficients of main interest are denoted in bold text.

Ψ Proxy firm ID was constructed using the unique combination of 5 variables we knew to vary at the firm level: the proportion of employees and dependents that were in an HDHP in the firm annually from 2016 through 2019 (4 variables) and firm industry.

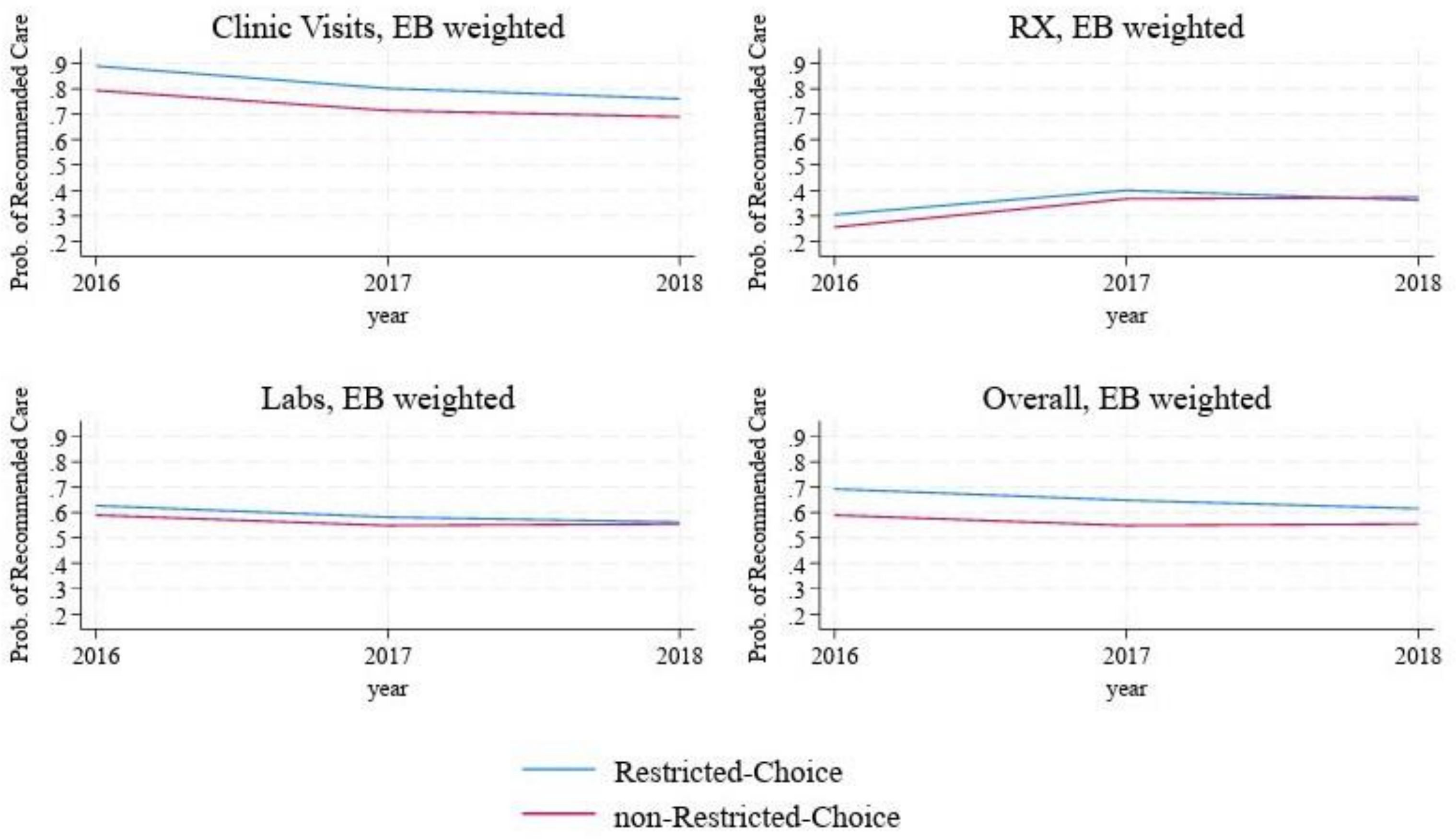
Clinic Visits, EB weighted
RX, EB weighted
Labs, EB weighted
Overall, EB weighted
Prob. of Recommended Care
year
.2
.3
.4
.5
.6
.7
.8
.9
2016
2017
2018
Restricted-Choice
non-Restricted-Choice
