# Supplementary material for: High-Deductible Health Plans and Receipt of Guideline-Concordant Care for Adults With Chronic Illness
Source: JAMA Netw Open. 2025 Apr 30;8(4):e258045. doi: 10.1001/jamanetworkopen.2025.8045 (PMC12044513; doi:10.1001/jamanetworkopen.2025.8045)
Supplement: Supplement 2. — Data Sharing Statement [file jamanetwopen-e258045-s002.pdf]

Data Sharing Statement

Gidwani. High-Deductible Health Plans and Receipt of Guideline-Concordant Care for Adults With Chronic Illness. *JAMA Netw Open*. Published April 30, 2025. doi:10.1001/jamanetworkopen.2025.8045

Data
Data available: No

Additional Information
Explanation for why data not available: The terms of the data use agreement with the data vendor (Merative MarketScan) do not allow us to re-share the data.
